# Immunomodulatory effects of new phytotherapy on human macrophages and TLR4- and TLR7/8-mediated viral-like inflammation in mice

**DOI:** 10.3389/fmed.2022.952977

**Published:** 2022-08-22

**Authors:** Olesia Schapovalova, Anna Gorlova, Johannes de Munter, Elisaveta Sheveleva, Mikhail Eropkin, Nikita Gorbunov, Michail Sicker, Aleksei Umriukhin, Sergiy Lyubchyk, Klaus-Peter Lesch, Tatyana Strekalova, Careen A. Schroeter

**Affiliations:** ^1^Caparica Faculdade de Ciencias e Tecnologia da Universidade Nova de Lisboa, NOVA Lisbon University, Lisbon, Portugal; ^2^Department of Psychiatry and Neuropsychology, School for Mental Health and Neuroscience, Maastricht University and Neuroplast BV, Maastricht, Netherlands; ^3^Laboratory of Psychiatric Neurobiology, Institute of Molecular Medicine and Department of Normal Physiology, Sechenov First Moscow State Medical University, Moscow, Russia; ^4^Laboratory of Cognitive Dysfunctions, Federal Budgetary Institute of General Pathology and Pathophysiology, Moscow, Russia; ^5^Department of Etiology and Epidemiology, Smorodintsev Research Institute of Influenza, St. Petersburg State University, Saint Petersburg, Russia; ^6^Division of Molecular Psychiatry, Center of Mental Health, University of Würzburg, Würzburg, Germany; ^7^Rehabilitation Research Unit of Clinic of Bad Kreuzbach, Bad Kreuzbach, Germany; ^8^EIGES Center, Universidade Lusofona, Lisboa, Portugal; ^9^Department of Pharmacology, University of Oxford, Oxford, United Kingdom; ^10^Preventive and Environmental Medicine, Cologne, Germany

**Keywords:** toll-like receptors, SARS-CoV-2, inflammation, pro-inflammatory cytokines, mice

## Abstract

**Background:**

While all efforts have been undertaken to propagate the vaccination and develop remedies against SARS-CoV-2, no satisfactory management of this infection is available yet. Moreover, poor availability of any preventive and treatment measures of SARS-CoV-2 in economically disadvantageous communities aggravates the course of the pandemic. Here, we studied a new immunomodulatory phytotherapy (IP), an extract of blackberry, chamomile, garlic, cloves, and elderberry as a potential low-cost solution for these problems given the reported efficacy of herbal medicine during the previous SARS virus outbreak.

**Methods:**

The key feature of SARS-CoV-2 infection, excessive inflammation, was studied in *in vitro* and *in vivo* assays under the application of the IP. First, changes in tumor-necrosis factor (TNF) and lnteurleukin-1 beta (IL-1β) concentrations were measured in a culture of human macrophages following the lipopolysaccharide (LPS) challenge and treatment with IP or prednisolone. Second, chronically IP-pre-treated CD-1 mice received an agonist of Toll-like receptors (TLR)-7/8 resiquimod and were examined for lung and spleen expression of pro-inflammatory cytokines and blood formula. Finally, chronically IP-pre-treated mice challenged with LPS injection were studied for “sickness” behavior. Additionally, the IP was analyzed using high-potency-liquid chromatography (HPLC)-high-resolution-mass-spectrometry (HRMS).

**Results:**

LPS-induced *in vitro* release of TNF and IL-1β was reduced by both treatments. The IP-treated mice displayed blunted over-expression of SAA-2, ACE-2, CXCL1, and CXCL10 and decreased changes in blood formula in response to an injection with resiquimod. The IP-treated mice injected with LPS showed normalized locomotion, anxiety, and exploration behaviors but not abnormal forced swimming. Isoquercitrin, choline, leucine, chlorogenic acid, and other constituents were identified by HPLC-HRMS and likely underlie the IP immunomodulatory effects.

**Conclusions:**

Herbal IP-therapy decreases inflammation and, partly, “sickness behavior,” suggesting its potency to combat SARS-CoV-2 infection first of all via its preventive effects.

## Introduction

Although the vaccination against SARS-CoV-2 is being implemented worldwide to curb the epidemic, none of the available vaccines provide full protection from the infection, and no specific drug to combat or prevent severe SARS-CoV-2 infection is anticipated (1, 2). As such, the search for alternative approaches to improve this situation has become the focus of much research (3). Excessive inflammation (i.e., the so-called “cytokine storm”), once established as a key pathophysiological feature of SARS-CoV-2 infection (4), became a target of the drug research and development in this area. The anti-inflammatory interventions were shown to be beneficial for both a prevention and treatment of viral infections, including SARS (4–6). These studies have resulted in the implementation of the preventive anti-inflammatory remedies and pathogenetic therapies in SARS-CoV-2 patients, such as hydroxychloroquine, chloroquine, azithromycin, ivermectin, colchicine, thalidomide and glucocorticoids methylprednisolone and dexamethasone, the monoclonal antibody tocilizumab, convalescent plasma interferons, and intravenous immunoglobulin therapy (7–9). However, these types of medicine are often unaffordable in low-income countries (10), which become a natural reservoir of the virus and a prerequisite of the appearance of new mutations (11).

The “cytokine storm” caused by SARS-CoV-2 can result in detrimental effects and even death (12–14). SARS-CoV-2 can activate the pattern recognition receptor (PRR) toll-like receptor (TLR) 4 that triggers the myeloid differentiation primary response (MyD) 88, causing a consequent NF-κB translocation to the nucleus and the upregulation of central and peripheral pro-inflammatory cytokines: tumor-necrosis factor (TNF), interleukin (IL)-1β, and IL-6 that increase the permeability of blood vessels and the migration of immune cells (15, 16). The SARS-CoV-2-induced inflammatory response also involves the IRF7-mediated TLR7/8 induction of type-1 interferon *via* a MyD88-dependent cascade and the upregulation of NFkB *via* the IL-1β receptor-associated kinase 1 (IRAK-1), IRAK-4, and TNF receptor-associated factor 6 (TRAF6) and of the type I interferons (IFNs) (15, 17). The activation of PRR toll-like receptors TLR4 and TLR7/8, *via* a series of molecular cascades, results in the upregulation of central and peripheral cytokines expression (18). It is regulated according to the nature of the pathogen and the TLR signaling pathways activated. Typically, TLR-mediated immune response involves an increase in circulating and central cytokines such as IL-1β, TNF and IL-6, as well as chemokines such as CXCL1, CCL2 and CXCL10 (15, 18, 19) and the induction of “sickness behavior,” i.e., reduced activity and exploration, and anxiety-like changes (15, 18–22).

The clinical management of “cytokine storm” is not a trivial challenge. For instance, the use of corticosteroids in SARS patients in 2003 increased mortality (23). Other therapies were not sufficiently effective either (24). At the same time, in the literature, the beneficial effects of herbal medicine combined with traditional medicine in SARS patients were demonstrated (4–6, 25–27), giving hope that therapeutically effective herbal compositions might combat the severe course of SARS-CoV-2. In addition, animal studies suggested possible mechanisms of anti-inflammatory immunomodulatory effects of medicinal herbs that target inflammatory pathways of TLRs-induced mechanisms, e.g., polysaccharides from red seaweed suppressed the expression of TNF, receptor-associated factor-6 in a model of LPS-induced toxicity (26), the use of vanilla extract suppressed free radical production in a mouse model of cancer (28, 29), ginger phenolics decreased lipid peroxidation and oxidative stress in rats (30). Our recent studies with a mouse ultrasound model of “emotional stress” have shown the beneficial action of herbal compositions with anti-inflammatory properties on the oxidative stress markers malondialdehyde and protein carbonyl and the expression of IL-1β and IL-6 (31, 32). Given important roles of excessive inflammation in the pathophysiology of severe course of SARS-CoV-2 infection, and beneficial effects of preventive and therapeutical application of herbal medicine with viral infections, we sought to study a preventive potential of a novel herbal composition that could be affordable as for instance in the communities with insufficient healthcare systems.

Therefore, we investigated the effects of a new immunomodulatory phytotherapy (IP), an extract of blackberry, chamomile, garlic, cloves, and elderberry (for the IP content, see Supplementary Table 1), that was designed as an anti-inflammatory composition (see Supplementary File) in previously established *in vitro* and *in vivo* models of inflammation (21, 22, 33). These paradigms were adapted from the classic experimental models that are based on the activation of TLRs, which implicate distinct but overlapping pathways (19). In particular, we recently established a model of peripheral inflammation that is induced by resiquimod, an agonist of TLR7/8 (33). In this model, strong up-regulation of IL-1β, TNF, IL-6, and chemokines in lungs, spleen, liver, and brain was decreased by the anti-inflammatory drug nafamostat (33). These recent studies showed that gene over-expression of SAA-2, ACE-2, CXCL1 and CXCL10 in the liver and spleen were effectively reduced by applied anti-inflammatory therapy and thus was investigated in the present work. We also used lipopolysaccharide (LPS), an agonist of TLR4, in an *in vitro* model of macrophage IL-1β and TNF release (19) and in an *in vivo* paradigm of “sickness behavior,” measuring inflammation-induced signs of anxiety, hypolocomotion, and suppressed exploration in mice (21, 22, 34, 35). High potency liquid chromatography (HPLC-HRMS) was employed to study the constituents of the IP.

## Methods

### Study flow

**Figure 1:**
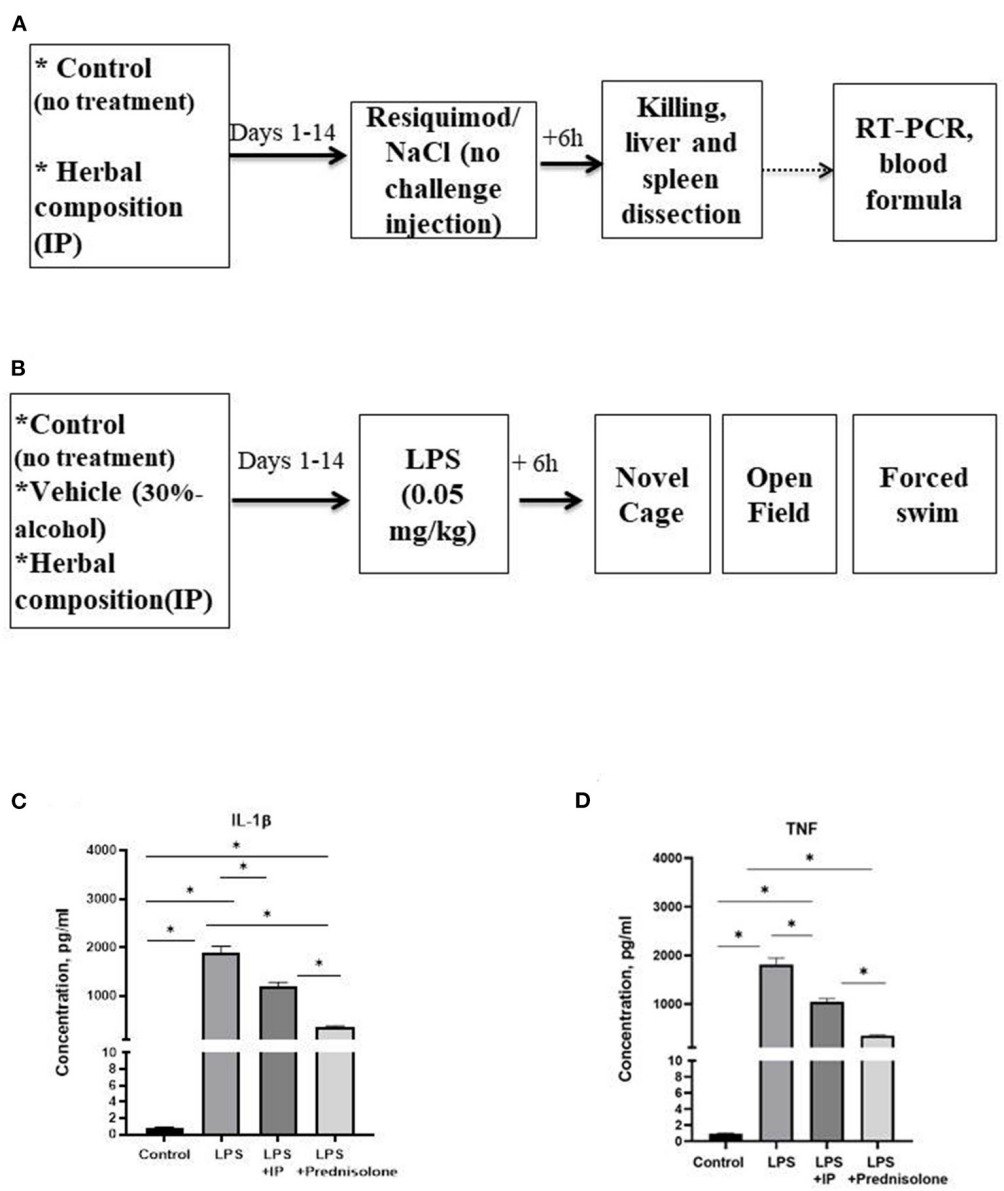
Schematic of *in vivo* tests performed and the outcome from the *in vitro* assay. In the *in vivo* experiments, animals were exposed to a daily administration of herbal drops or vehicle during the 14 days. On the day of the experiment, mice received an intraperitoneal injection **(A)** with resiquimod or vehicle and killed 6 h post-challenge, **(B)** with LPS or vehicle and 6 h post-challenge completed the novel cage, open field, and the forced swim test. In the *in vitro* assay, herbal drops or prednisolone was applied in the human macrophage cell culture that was treated with LPS. In comparison with the non-stimulated samples, there was a significant increase in the concentrations of **(C)** IL-1β and **(D)** TNF in all LPS-challenged samples. Significant group differences: *vs. non-challenged samples, (one-way ANOVA and Tukey's test). Data presented as mean ± SEM.

In the *in vitro* study, we studied the release of TNF and IL-1β by LPS-challenged human macrophages that were pre-treated with IP or prednisolone, about 10 samples were used per condition. We next pre-treated CD-1 mice with an IP herbal composition for 2 weeks (36) and intraperitoneally injected them with resiquimod (200 μg). Mice (*n* = 7–8 in each group) were culled 6 h post-challenge and examined for liver and spleen genes mRNA concentrations of inflammatory markers whose expression was most profoundly altered in our previous study: SAA-2, ACE-2, CXCL1, CXCL10, IL-1β, IL-6, and for blood formula (33) (Figure 1A). Finally, using the same IP dosing conditions, pre-treated CD-1 mice were challenged with a low dose of LPS (0.05 mg/kg) and 6 h post-challenge were investigated for helplessness, locomotion, anxiety-like, and exploratory behaviors in the open field, novel cage, and forced swim models (*n* = 6–7 in each group, Figure 1B). Separately, high potency liquid chromatography (HPLC)-high resolution-mass-spectrometry (HRMS) was employed to analyze biologically active constituents of the IP. All experiments were approved by the University of Oxford local committees (LERP, ACER) in accordance with the UK Animals (Scientific Procedures) Act 1989 and iCell2 METC Zuyderland Zuid, the Netherlands and MSMU#11-18-2018/2019 and were compliant with ARRIVE guidelines (http://www.nc3rs.org.uk/arrive-guidelines).

Experiments were performed on male 2.5-months-old CD-1 male mice that were purchased by a provider licensed by Charles River (http://www.spf-animals.ru/about/providers/animals). Mice were housed under standard conditions (in plastic cages 27 cm × 22 cm × 15 cm, 22 ± 1°C, 55% humidity, food and water *ad libitum*), reversed 12-h light/dark cycle (lights on at 19:00). All efforts were undertaken to minimize the potential discomfort of the animals.

### A study of LPS-induced cytokine release by human macrophages

Human macrophages from healthy volunteers of both sexes were used. The effects of the IP application on the LPS-stimulated release of IL-1β and TNF were determined and compared against potential effects of the IP on non-stimulated macrophages, the effects of a standard anti-inflammatory treatment with prednisolone, and a release of non-treated LPS-challenged macrophages (see Supplementary File). Separate studies that were carried out to rule out potential effects of the IP-alcohol-containing vehicle on these read-outs showed a lack of such effects (Supplementary Figure 1).

### Induction of systemic inflammation in mice

The first cohort of mice received an intraperitoneal (i.p.) injection of resiquimod (R848, Enzo Life Sciences, Farmingdale, NY, USA) that was diluted in a DMSO-vehicle (1 mg/mL). Because the administration of DMSO-vehicle alone did not alter the immunological response (37), it was not used in the present study. The second cohort of mice was treated with an i.p. injection of LPS (0.05 mg/kg, *E.coli* 0111:B6, Sigma-Aldrich, Gillingham, UK) dissolved in NaCl (21, 22, 36). The choice of this dose was based on separate control studies showing the “ceiling” behavioral changes in mice injected with the LPS dose of 0.1 mg/kg (see Supplementary Figure 2).

### The administration of the IP

The drops of the IP were administered orally (120 μL per day) for each mouse during the morning hours using a pipette (26, 36). A composition of a 30% alcohol IP solution can be found in a Supplementary Table 1.

### Behavior

To determine the effect of the IP on the LPS-induced “sickness” behavior, novel cage, open field, and forced swim tests were performed (38), see Supplementary File.

### Tissue collection

Mice were anesthetized with isoflurane (21, 22). Blood (200 μL) was then collected by cardiac puncture, transferred into an EDTA-coated tube, and immediately analyzed for blood formula. Animals were then intracardially perfused with cold saline, liver and spleen were collected and snap-frozen.

### Blood analysis

Blood was measured in triplicate on the ABX Pentra 60 (Horiba, Northampton, UK). The number of lymphocytes, monocytes, neutrophils, basophils, and eosinophils per μL (cells/μL) and percent of the cells were counted (33).

### RNA extraction, CDNA conversion and QPCR

According to the manufacturer's instructions, RNA was extracted from samples of liver and spleen using the Qiagen RNeasy Mini kit, RNA concentration was measured using a NanoDrop, and 1,000 ng of RNA was converted to cDNA with the Applied Biosystems High Capacity cDNA conversion kit. Real-time qPCR was performed with samples in duplicate (25 ng/well) using the SYBR green qPCR master mix (PrimerDesign, Camberley, UK) with the Roche LightCycler 480 (33). Relative expression was determined by the 2-ΔΔCT method, normalized to GAPDH as the housekeeping gene (PrimerDesign, Camberley, UK); for the list of primers, see Supplementary Table 2.

### High-potency-liquid chromatography high-resolution-mass-spectrometry

The solution was analyzed using the chromatographic system Agilent 1,290 Infinity II, quadrupole-time streaming high precision mass detector Agilent 6,545 Q-TOF LC/MS, and Zorbax Eclipse Plus C18 RRHD columns (Agilent Technologies, Santa Clara, CA, USA); for details, see Supplementary File.

### Statistical analysis

Statistical analyses were performed with the GraphPad Prism 7 software. Data sets were tested for normal distribution; Welch's test and *t-test* were used to perform two group comparisons where appropriate, and one or two-way analysis of variance (ANOVA) was employed for multiple group analysis, with Tukey's *post-hoc* test. Results were considered significant at *p* < 0.05 with 95% confidence intervals. In the study with resiquimod, data were expressed as percent of challenged groups from the respective non-challenged groups that either received the IP or were not treated with the immunomodulatory agent and were compared to a 100%-level. Data are expressed as mean ± standard error of the mean (SEM).

## Results

### Application of the IP reduces cytokine release from LPS-induced macrophages

The IL-1β and TNF concentrations in the macrophage cell culture were significantly different between the groups (*F* = 145.6 and *F* = 94.45, respectively, both *p* < 0.0001, one-way ANOVA). In the challenged non-treated samples, there were significant increases in these parameters compared to the non-treated group, as well as to the LPS-challenged samples treated with the IP or prednisolone (all *p* < 0.0001, Tukey's test, Figures 1C,D). The IL-1β and TNF levels were significantly higher in both the IP- and prednisolone-treated preparations than in the non-treated samples (IL-1β: *p* = 0.0021 and *p* < 0.0001, respectively, TNF: both *p* < 0.0001), whereas the latter groups had lower IL-1β concentrations compared with the IP-treated group (IL-1β: *p* < 0.0001; TNF: *p* = 0.0001).

### Chronic administration of the IP diminishes the resiquimod-induced expression of inflammatory markers in the liver and spleen

**Figure 2:**
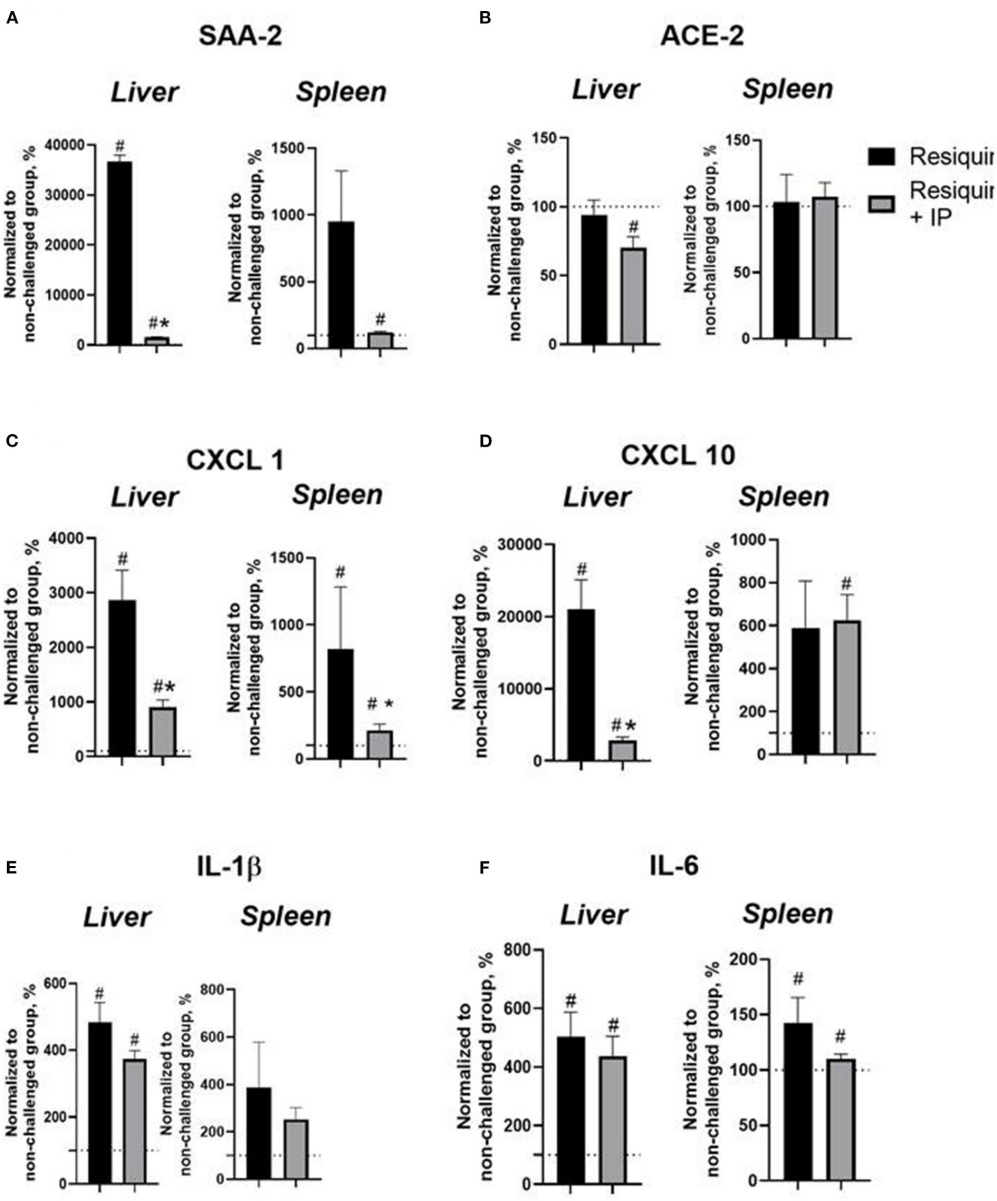
Pro-inflammatory resiquimod-induced gene expression changes in the liver and spleen are ameliorated with the herbal IP treatment. **(A)** Resiquimod-challenged groups showed a significant increase in the normalized liver expression of SAA-2mRNA in comparison to a 100%-level. This measure was lower in the liver of IP–treated animals. No such differences were shown for the spleen. **(B)** As compared to a 100%-level, there was a significant decrease in the normalized ACE-2mRNA expression in the liver of the IP-treated group but not in the non-treated resiquimod-challenged mice that was not found for the spleen **(C)** Both resiquimod-challenged groups showed a significant increase in liver CXCL1mRNA normalized concentrations as compared to 100%; the IP-treated group subjected to the resiquimod injection had a significantly decreased normalized liver CXCL1mRNA expression compared with resiquimod-injected animals. In the spleen, compared to 100%, this measure was decreased in the IP-treated resiquimod-challenged group. **(D)** We found a significant increase in the normalized CXCL10mRNA expression in the liver and spleen compared to 100% in both groups, while this measure was lower in the IP-treated group than in resiquimod-challenged mice without treatment; no group difference in this measure was found for the spleen. **(E)** Elevated normalized IL-1βmRNA levels compared to 100% in the liver and spleen were shown for both groups; no other differences were found. **(F)** Normalized mRNA expression of IL-6 was similarly elevated in two groups in the liver and spleen compared to the 100% level. No other differences were found. *vs. 100% level, # vs. the challenged non-treated group (Welch's test and *t*-test, see the text). Data presented as mean ± SEM.

All non-normalized to unchallenged values can be found in the Supplementary Tables 3, 4. In the liver, a comparison of the resiquimod-challenged groups showed that the normalized SAA-2mRNA expression in the IP-treated mice was lower than in the non-treated mice (*p* < 0.0001, *Welch's test*, Figure 2A); compared to 100%, both groups had an elevated SAA-2mRNA expression (*p* < 0.0001). Both challenged groups demonstrated an increased normalized SAA-2mRNA expression in the spleen (*p* = 0.0137, vs. 100%, Figure 2A). There was a trend of a lower normalized ACE-2mRNA expression in the IP-treated group than in the resiquimod-challenged non-treated mice (*p* = 0.093, Figure 2B) and a significant decrease in this parameter in the former but not the latter group as compared to 100% (*p* = 0.005 and *p* = 0.594, respectively). No group differences were found in the normalized spleen ACE-2mRNA expression level (*p* = 0.874, Figure 2B). Compared to 100%, no significant difference was observed in any group (*p* = 0.877 and *p* = 0.525, respectively).

The IP-treated group subjected to the resiquimod injection had a significantly decreased normalized liver CXCL1mRNA expression compared with the resiquimod-injected animals (*p* = 0.0054, Figure 2C), while both groups showed a significant increase in this measure as compared to 100% (*p* = 0.0006 and *p* = 0.0001, respectively). No group differences in the normalized CXCL1mRNA expression in the spleen were found (*p* = 0.372, Figure 2C). However, compared to 100%, the mRNA expression level in the IP-treated resiquimod-challenged group was diminished (*p* = 0.034) with no difference in mice that received the resiquimod alone (*p* = 0.137).

A significant decrease in the normalized CXCL10mRNA expression in the liver was observed for the IP-treated mice with induced inflammation compared with mice treated with resiquimod alone (*p* = 0.0016, Figure 2D). Compared to 100%, the mRNA liver expression of this gene was elevated in both groups (*p* = 0.0006 and *p* = 0.0001, respectively). In the spleen, no group difference in this parameter was found (*p* = 0.885, Figure 2D). Both groups revealed an elevated CXCL10mRNA expression compared to 100% (*p* = 0.047 and *p* = 0.002, respectively).

As for the normalized mRNA concentration of IL-1β in the liver, there was a trend of a decreasing expression level in the IP-treated mice with induced inflammation compared to the resiquimod- challenged group (*p* = 0.111, Figure 2E). Elevated IL-1βmRNA levels compared to 100% were shown for both groups (*p* < 0.0001). No significant group differences were shown in this parameter in the spleen (*p* = 0.473, Figure 2E); IL-1βmRNA expression was elevated compared to 100% in both groups (*p* = 0.014 and *p* = 0.0079, respectively). The normalized mRNA expression of IL-6 was similar in two groups in the liver and spleen (*p* = 0.553 and *p* = 0.252, respectively, Figure 2F) and was elevated compared to 100% in both groups (liver: *p* = 0.001 and *p* = 0.0008; spleen: *p* = 0.028 and *p* = 0.037, respectively).

### The effects of the IP on the resiquimod-induced changes in the blood formula

**Figure 3:**
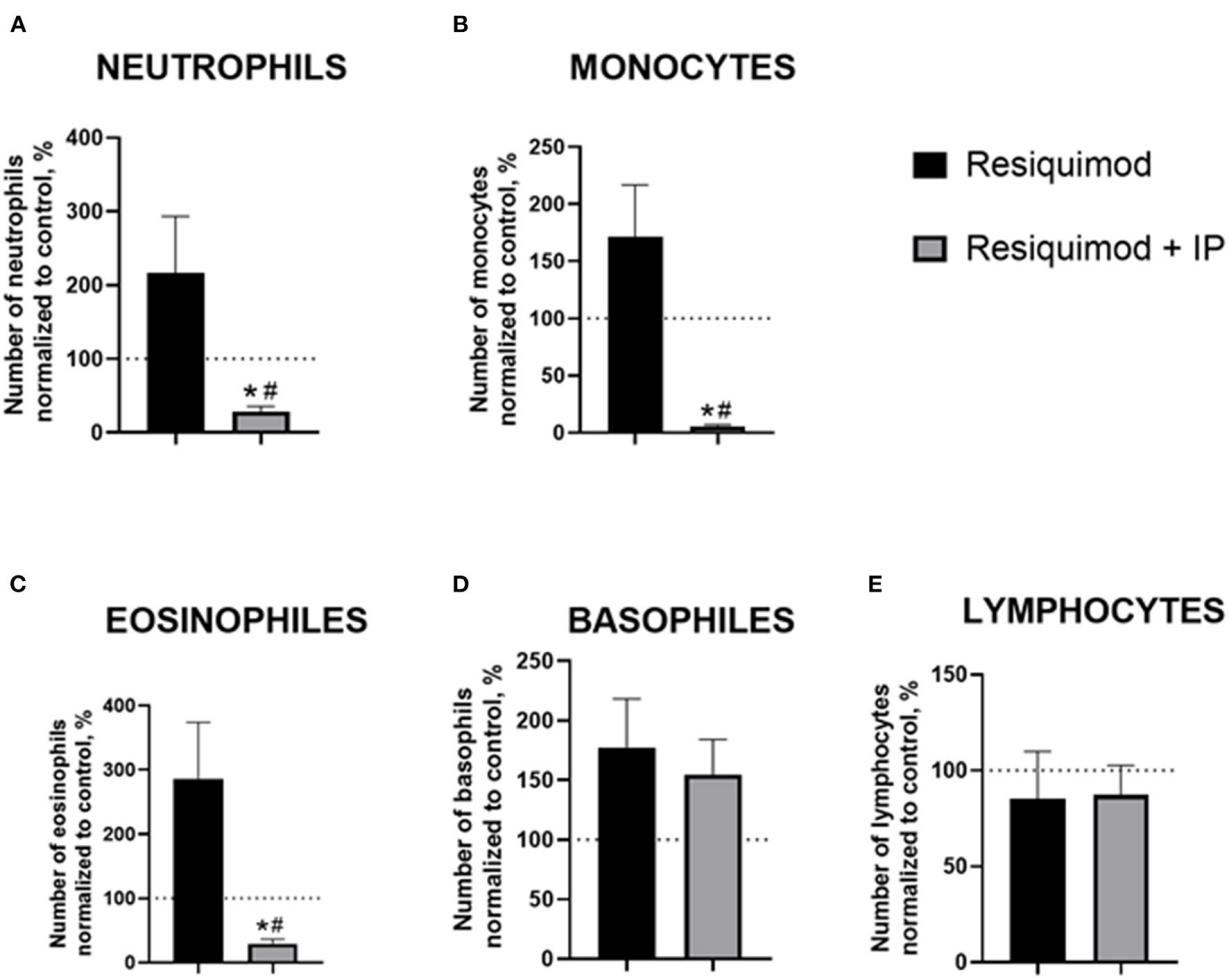
Pro-inflammatory effects of resiquimod on blood formula are reduced in mice treated with the herbal IP composition. Normalized counts of **(A)** neutrophils, **(B)** monocytes, **(C)** eosinophils were significantly decreased in IP-treated resiquimod-stimulated mice. **(D)** An increase in basophil counts was similar in the two challenged groups. **(E)** No changes were found in the lymphocyte counts of the resiquimod-injected groups; *vs. 100% level, #vs. the challenged non-treated group (Welch's test and *t*-test, see the text). Data presented as mean ± SEM.

All non-normalized to unchallenged values can be found in the Supplementary Table 5. In the resiquimod-challenged non-treated animals, there was a trend of elevated counts of blood neutrophils, monocytes, and eosinophils compared to a 100%-level (neutrophils: *p* = 0.165, monocytes: *p* = 0.151, eosinophils: *p* = 0.066, *t-test*), while the IP-pre-treated challenged mice showed opposite changes (all *p* < 0.0001, Figures 3A–C). The latter group revealed a significant decrease in all counts compared to the resiquimod-injected animals (neutrophils: *p* = 0.038; monocytes: *p* = 0.006; eosinophils: *p* = 0.019).

Both the non-treated- and IP-treated mice challenged with resiquimod had similar non-significant increases in basophils compared with a 100% level (*p* = 0.094 and *p* = 0.099, respectively, Figure 3D). No differences were found between the challenged groups (*p* = 0.655). The number of lymphocytes did not differ between the resiquimod-treated groups and a 100%-level (*p* = 0.564 and *p* = 0.432, respectively, Figure 3E), nor did it differ between the groups (*p* = 0.941).

### Effects of the IP on the LPS-induced “sickness” behavior

**Figure 4:**
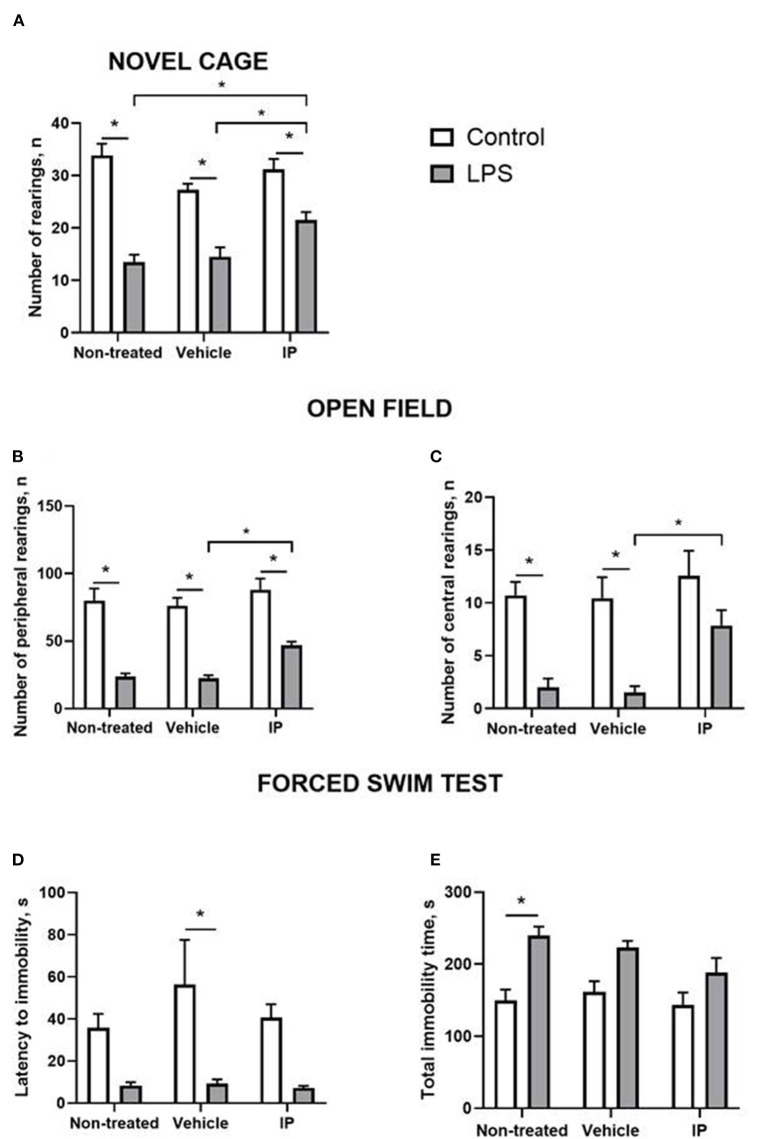
LPS induces a sickness behavior phenotype, which is partly normalized by the herbal IP. In comparison to non-treated challenged animals, IP-treated LPS-challenged mice displayed **(A)** a normalized number of exploratory rears, **(B)** a number of peripheral and **(C)** central crossings in the open field (two-way ANOVA and Tukey's test). There were no such differences **(D)** in the latency to float and **(E)** duration of the floating in the forced swim test; *vs. the challenged non-treated group. Data presented as mean ± SEM.

In the novel cage test, two-way ANOVA demonstrated a significant LPS effect on the number of exploratory rearings (*F* = 103.0, *p* < 0.0001). There was a significant treatment effect and LPS x treatment interaction (*F* = 4.928, *p* = 0.0125 and *F* = 5.093, *p* = 0.011, respectively). The *post-hoc* test showed that this measure was significantly smaller in the Vehicle LPS group and IP-treated LPS group compared to the corresponding control groups (*p* < 0.0001; *p* < 0.0001 and *p* = 0.0053, respectively, Tukey's test, Figure 4A). The IP-treated group with LPS-induced inflammation had a significantly higher number of rearings compared with the non-treated LPS group and Vehicle LPS group (*p* = 0.016 and *p* = 0.048, respectively, Figure 4A).

Both significant LPS and treatment effects were revealed by two-way ANOVA in the open field test (*F* = 125.8, *p* < 0.0001 and *F* = 6.357, *p* = 0.004, respectively). The number of peripheral rearings was lower in the non-treated LPS group, Vehicle LPS group, and IP-treated LPS group compared to the corresponding control groups (*p* < 0.0001; *p* < 0.0001 and *p* = 0.0001, respectively, Figure 4B). The IP-treated group with LPS-induced inflammation had a significantly higher number of rearings compared with the Vehicle LPS group (*p* = 0.028, Figure 4B).

The two-way ANOVA showed significant LPS and treatment effects in the number of central rearings in the open field test (*F* = 37.31, *p* < 0.0001 and *F* = 4.837, *p* = 0.0134, respectively). This parameter was lower in the non-treated LPS group and Vehicle LPS group compared to the corresponding control groups (both *p* = 0.002, Figure 4C). The number of central rearings in the IP-treated group with LPS induced inflammation was significantly higher compared to the Vehicle LPS group (*p* = 0.028, Figure 4C).

The two-way ANOVA revealed the LPS effect in the latency of immobility in the forced swim test (*F* = 23.84, *p* < 0.0001). This measure was higher in the control Vehicle group compared to the Vehicle group with LPS-induced inflammation (*p* = 0.007, Figure 4D). The two-way ANOVA showed a significant LPS effect in the total time of immobility in this test (*F* = 27.25, *p* < 0.0001). The total time of immobility was higher in the non-treated LPS group compared to the corresponding control group (*p* = 0.002, Figure 4E). No other significant effects were found.

Thus, chronic IP administration can attenuate some but not all signs of “sickness” behavior caused by the LPS-induced systemic inflammation, whereas the alcohol-containing vehicle does not generate these effects.

### HPLC-HRMS

HPLC-HRMS revealed bioactive constituents of the IP: choline, γ-Glutamyl-(S)-allyl-cysteine, N-Fructosyl or glucosyl isoleucine, L-glutamyl–L-phenylalanine (Glu-Phe), chlorogenic acid, phenylalanine, tryptophan, isoleucine, syringin, and isoquercitin (see Supplementary Table 6). The following meta-analysis showed that these components were previously reported to modulate the immune response and inflammation, as well as oxidative and nitrosative stress, and thus, are likely to underlie the reported immunomodulatory properties of the investigated herbal IP (see Supplementary Table 7). The outcome from the meta-analysis study and the main effects of these eleven elements are reviewed in the Supplementary File.

## Discussion

Here, we have shown that the administration of the IP herbal composition containing an extract of blackberry, chamomile, garlic, cloves, and elderberry can reduce pro-inflammatory changes that are reminiscent to a “cytokine storm,” a key feature of SARS-CoV2 infection. These *in vitro* and *in vivo* effects were demonstrated in the established models of systemic inflammation that are based on triggering of TLR7/8 and TLR4, the key mediators of this pathological condition. While the effects on molecular and behavioral markers of response to inflammatory challenges were suppressed partially, this herbal composition can be efficient in diminishing deleterious manifestations of the “cytokine storm” caused by the SARS-CoV2 virus, at least, in a preventive manner.

The anti-inflammatory effect of the IP was first evaluated by measuring *in vitro* the release of IL-1β and TNF by LPS-challenged macrophages and compared to that of prednisolone. For both cytokines, their release was suppressed by the IP, though the effect of prednisolone was much greater. IL-1β and TNF are inflammatory mediators that have long been associated with the “cytokine storm” caused by SARS-CoV-2 (7, 39, 40). TLRs are strongly expressed in macrophages (41), and it is seems probable that the IP counteracts their activation caused by LPS (42).

The present study revealed the suppressive effect of the IP administration on TLR7/8-mediated inflammation caused by resiquimod. The IP decreased the counts of neutrophils, monocytes, and eosinophils that were elevated by the injection of resiquimod, though the number of basophils was increased regardless of the pre-treatment with the IP. Increases in these blood cell counts in response to the resiquimod administration are considered characteristic signs of excessive immune activation (43–45). A suppression of these responses by the IP administration demonstrates its anti-inflammatory properties, which were shown in this model for the anti-inflammatory treatment with nafamostat (33). These former experiments have validated the applied here model as pharmacologically sensitive to anti-inflammatory interventions. As such, the reported here effects of IP on blood formula can be interpreted to be similar to that of pharmacological anti-inflammatory reference. Previous studies also revealed resiquimod-induced lymphopenia in CD-1 mice, more specifically, a decrease in the counts of circulating lymphocytes recapitulating clinical and experimental observations of acute viral infection (46–48), including after TLR7 stimulation (49) that was also found in SARS-CoV-2 patients (22, 50). In the present study, a decrease in lymphocytes did not reach a level of significance.

The magnitude of the systemic inflammatory response was evaluated by measuring the relative expression of pro-inflammatory genes in the liver and spleen (22, 51, 52). In both organs, the resiquimod challenge induced a significant increase in SAA-2, ACE-2, CXCL1, and CXCL10. These inflammatory mediators were established to accompany viral infection and underlie sickness behavior (40), and were shown to be significantly up-regulated in our previous study with resiquimod (33). TLR7 is strongly expressed in macrophages, and it seems probable that it is the resident tissue macrophages that are responsible for producing these cytokines (42), which would also account for the differential expression levels between the organs, as there is a greater density of macrophages in the liver than the spleen. Here, we were also able to show that the herbal IP had a peripheral anti-inflammatory effect; hepatic SAA-2, CXCL1, and CXCL10 expression induced by resiquimod was significantly ameliorated by the IP treatment, as well as ACE-2, to a lesser extent. As for IL-1β and IL-6, there was just an optical trend for the normalizing effects of the IP. A suppression of these changes in gene expression by the IP administration evidences its anti-inflammatory properties, which were demonstrated in this model for nafamostat (33). Because the SARS-CoV2 virus needs to bind to the host ACE2 receptor *via* its spike protein to infect cells, its suppression by the IP shown in our work let to speculate it can be considered a potential prevention remedy.

In our study, locomotion, anxiety-like, and exploratory behavior in mice were significantly affected by the injection of a low dose of LPS, which is consistent with other inflammatory models such as those employing the classical LPS-CD14-TLR4 challenge (21, 34). Analysis of central crossings in the open field revealed that the LPS-treated animals chose to spend less time in the center of the open field, which suggests that LPS had an anxiogenic effect. Increased anxiety has been reported in LPS models (22, 35) and is associated with the central expression of pro-inflammatory cytokines. In a view of previously reported inter-relation between behavioral and molecular effects of LPS in models of systemic inflammation in mice (21, 22), reported here behavioral effects of the administration of IP can be interpreted as a manifestation of its anti-inflammatory action.

Of note, the behavioral effects of the IP on LPS-challenged mice were partial, as it did not reach a statistical significance in the measures of helplessness during the forced swimming. However, forced swimming in rodents is not often associated with the “sickness” behavior; previous studies with an LPS challenge were unable to demonstrate consistent changes in this test (53). Collectively, for the IP, the behavioral changes observed are indicative of a “sickness” behavior phenotype and were overly reduced by chronic administration.

Importantly, the present study showed that the IP herbal composition has ameliorated the LPS-induced *in vitro* cytokine release and “sickness behavior” that was not altered by alcohol vehicle alone, while alcohol might have subtle anti-inflammatory effects (54, 55). As the inhibition of cytokine signaling in the periphery can attenuate “sickness behaviors” induced by the injection of IL-1β (56, 57), the suppression of the inflammatory response in liver and spleen in IP-treated animals that is reported here can explain the beneficial behavioral effects of chronic pre-treatment with the employed herbal composition.

Our work has used CD-1 mice as a mouse strain that is highly susceptible to inflammatory challenges in comparison with other mouse lines, as some studies suggest (58). While strain differences were shown to affect the response to a systemic inflammation (58, 59), overly similar molecular and behavioral changes that were reported in C57Bl6 mice, Balb/c and CD-1 mice following pro-inflammatory challenges (58–60) suggest that reported here findings are unlikely to be strain-specific.

Because the pathophysiology of SARS-CoV2 comprises the mechanisms of viral invasion and replication that are SARS-CoV2-specific, as well as excessive, uncontrolled inflammation, which can be a common element of any severe infection, the IP can be regarded as a useful non-specific preventive remedy of this infection. Notably, while the present study was not designed to address the question whether or not, similarly to other herbal medicine, the administration of IP can exert disease-specific therapeutic action (4, 5), its significant effects on highly up-regulated inflammatory markers and expression of ACE-2 mediating the viral binding in the host may suggest such possibility. Further experiments are required to test this hypothesis.

HPLC-HRMS analysis has revealed the main constituents of the IP that likely underlie its beneficial immunomodulatory effects: choline, γ-Glutamyl-(S)-allyl-cysteine, N-Fructosyl or glucosyl isoleucine, L-glutamyl–L-phenylalanine (Glu-Phe), chlorogenic acid, phenylalanine, tryptophan, isoleucine, syringin, and isoquercitin. Choline is a well-established important macronutrient that regulates synaptic plasticity, e.g., *via* several genes, G9a, Prmt1, Ahcy, Dnmt1, Mat2a (61, 62), implicated in neurotrophic processes (63), potentially *via* insulin-like-growth-factor-2 (IGF2) and insulin receptor-mediated mechanisms (64, 65). Of note, the activation of insulin receptor mediated signaling triggers anti-inflammatory cascades (66). Among other activities, γ-Glutamyl-(S)-allyl-cysteine was shown to stabilize radical-scavenging and metal-chelating processes that are important in immune responses and contribute to the immunological regulation of the IP (67). The ability of N-Fructosyl or glucosyl-isoleucine to regulate the mechanisms of stress response has been documented (68). Another IP component, L-glutamyl–L-phenylalanine, was found to diminish liver inflammation *via* reducing lipid accumulation and presumably acting on metabolic processes of the cell (69). Tryptophan, a molecule with the greatest anti-oxidative capacity among amino acids (70) and a precursor of the neurotransmitter serotonin that is known to exert anti-inflammatory action (71), was shown to act *via* calcium-dependent mechanisms of receptor activation (72).

Moreover, the IP contains chlorogenic acid whose anti-inflammatory effects are well-documented in a model of transient forebrain ischemia and associated with a reduction in the levels of pro-inflammatory factors: SOD2, IL-2, TNF and an increase in the expression of anti-inflammatory cytokines: IL-4, IL-13 (73), resulting in anti-oxidative action also *via* the activation of antioxidant enzymes and neuroprotective effects. This spectrum of activities was further demonstrated in rat models of ischemia/re-perfusion of the kidney and liver (74–76). Another IP constituent with documented anti-oxidative stress effects is phenylalanine (77, 78), which was shown to decrease the production of reactive oxygen species (78, 79). Similarly, isoleucine counteracts the mechanisms of oxidative stress, as shown in a model of H_2_O_2_-stimulated intestinal epithelial cells (80), ameliorates NO-mediated pathways during wound healing (81), and exerts an immunomodulatory action regulating the key molecules of the mammalian innate immunity, β-defensin (82). Finally, isoquercitin is one of the most powerful well-studied natural anti-inflammatory and anti-oxidant agents that acts directly on the scavenging of reactive oxygen/nitrogen species (83), inhibits production of pro-inflammatory cytokines, pro-oxidant enzymes, and prostaglandins (83, 84), and induces antioxidant enzymes (85–87).

## Conclusion

Critically, the over-expression of inflammatory markers, blood cell inflammatory response, and “sickness behavior” under conditions of strong pro-inflammatory stimuli were ameliorated by the treatment with the herbal composition IP. Given that the magnitude of these responses was associated with disease severity (39, 87) and anti-inflammatory treatment (88), our findings suggest that the IP preventive treatment may reduce the severity of SARS-CoV2 infection. Since our results were achieved in the absence of viral entry, we propose that the IP may have generally useful anti-inflammatory effects, which may be advantageous in the treatment of various viral infections, including SARS-CoV2. The effect of the new immunomodulatory phytotherapeutic herbal extract is required further evaluation on patients with SARS-CoV-2 infection. Therefore, it is advocated to recommend a future clinical randomized controlled trial study that implement the conventional treatment with herbal extracts. As highlighted previously, the availability of low-cost herbal medicine for economically disadvantageous communities is of particular importance to curb SARS-CoV2 pandemic and enhancing preparedness for future pandemics.

## Data availability statement

The raw data supporting the conclusions of this article will be made available by the authors, without undue reservation.

## Ethics statement

The animal study was reviewed and approved by University of Oxford Local committees LEPR and ACER, iCell2 METC Zyaderland Zuid, the Netherlands.

## Author contributions

CS, TS, SL, and AU conceived the study. JM, SL, TS, and ME designed the experiments. OS, AG, NG, and MS carried out the experiments, data analysis, and performed the literature study. OS, JM, MS, ME, and AG performed the graph preparation and statistical analyses. K-PL, CS, and TS supervised the project. SL, K-PL, TS, and CS got the funding. CS, OS, AG, AU, NG, and TS wrote the initial draft of the manuscript and all other authors listed here revised it. All authors contributed to the article and approved the submitted version.

## Funding

This study was supported by Eat2beNice EU framework (2018-2023, to TS and K-PL) and by PhytoAPP EU framework (2021-2025, to OS, SL, and TS). The Eat2beNICE project has received funding from the European Union's Horizon 2020 research and innovation programme under grant agreement No 728018 and the PhytoAPP project has received funding from the the European Union's HORIZON 2020 research and innovation programme under the Marie Sklodowvska-Curie grant agreement 101007642. This publication reflects only the author's views and the European Commission is not liable for any use that may be made of the information contained therein. This publication was supported by the Open Access Publication Fund of the University of Wuerzburg. NG was supported by the German Research Foundation (DFG RTG 2660 under grant No. 433490190).

## Conflict of interest

The authors declare that the research was conducted in the absence of any commercial or financial relationships that could be construed as a potential conflict of interest.

## Publisher's note

All claims expressed in this article are solely those of the authors and do not necessarily represent those of their affiliated organizations, or those of the publisher, the editors and the reviewers. Any product that may be evaluated in this article, or claim that may be made by its manufacturer, is not guaranteed or endorsed by the publisher.
