# Supplementary material for: Immunomodulatory effects of new phytotherapy on human macrophages and TLR4- and TLR7/8-mediated viral-like inflammation in mice
Source: Front Med (Lausanne). 2022 Aug 22;9:952977. doi: 10.3389/fmed.2022.952977 (PMC9450044; doi:10.3389/fmed.2022.952977)
Supplement: Supplementary file 1 [file Data_Sheet_1.pdf]

Supplementary file

Composition of herbal immunomodulatory phototherapeutic (IP)

The drops are made out of herbs, spices and of dried fruits (see *Supplementary Table 1*) diluted in 30% alcohol solvent (kindly provided by R. Vendeville, Voorhout, The Netherlands).

Supplementary Table 1. Composition of IP and IP alcohol-containing solvent

Solution	SHC	SHC-solvent
**Composition**	Blackberry<br>Chamomile<br>Garlic<br>Clove<br>Elderberry<br>Alcohol 30%<br>water	Alcohol 30%,<br>water

A new immunomodulatory phototherapeutic IP was designed as an anti-inflammatory composition, as its herbal elements were previously shown to reduce the inflammation in clinical and pre-clinical studies (Nunes et al. 2020; Nascimento et al., 2021). For example, garlic is well established as an immunomodulatory and anti-inflammatory agent (Batiha et al., 2020); the consumption of aged garlic extract modulated the distribution of immune cells and prevented an increase in the concentration of TNF and IL-6 in a serum in obese adults (Xu et al., 2018). Lee et al. found that the chloroform extract from aged black garlic reduces TNF–induced NF-κB activation in human umbilical vein endothelial cells (Jeong et al., 2016). Moreover, in the double blind randomized clinical trial the administration of 400 mg of standardized garlic extract resulted in a significant reduction in IL-6, CRP and ESR blood levels (Zare et al., 2019).

The application of vanilla extract was shown to suppress free radical production in a mouse model of cancer (Tai et al., 2011). Elderberry was shown to affect inflammatory markers, although this effect did decline with an ongoing supplementation and it were weaker than that of classic non-steroid ant-inflammatory drugs (Vogl et al., 2013; Wieland et al., 2021). Blackberry was shown to suppress inflammatory response of macrophages (Cenk et al., 2021) and was proposed as a component of medical food with anti-inflammatory properties (Paczkowska-Walendowska et al., 2021).

Extensive literature suggests therapeutic potential of clove, *Syzygium aromaticum*, that is used in traditional medicine to treat inflammation of the mouth and throat, nausea and upset stomach (Han and Parker, 2017). Synergistic interactions between non-steroidal anti-inflammatory drugs and *Matricaria chamomilla* extract were shown to mediate anti-inflammatory effects at the systemic level that might be of therapeutic benefits for the clinical management of inflammatory processes (Ortiz et al., 2017).

However, many studies suggest that the use of herbal combinations might be more effective in their anti-inflammatory and ant-oxidant action, in comparison with the effects of an individual use of these and other medicinal herbs (Xia et al., 2021).

LPS-induced cytokine release by human macrophages

Blood was collected from 42 healthy volunteers and samples of a volume of 9 ml were subjected to a heparinization. Subsequently, the samples were diluted 1:10 with RPMI 1640 medium (ThermoFisher, Gibco, Hennigsdorf, Germany) and were cultured for 6 hours, at the temperature 37º, at 5% CO_2, 96%-humidity, with and without LPS (Lipopolysaccharide E. coli, Merck, Darmstadt, Germany) that was used at the concentration of 25 ng/ml. Cell preparations were treated with prednisolone that was applied at its final concentration of 2,5µg/ml, or with IP that was used that the final concentration of a 38% v/v, or remained untreated. Supernatants were collected and concentrations of IL-1β and TNF were determined using flow cytometry according to BD Cytometric Bead Array (CBA). Human Soluble Protein Master Buffer Kit (Becton Dickinson, Franklin Lakes, NJ, USA) was used according the manufacturer instructions.

Lack of effects of alcohol-containing vehicle on the LPS-induced cytokine release

Separate pilot studies were carried out to rule out potential effects of the IP-alcohol-containing vehicle on the cytokine release and showed a lack of such effects (*Supplementary Fig.1*). A 30% alcohol-containing vehicle was applied as described above, instead of IP. One-way ANOVA revealed significant group differences in the study with non-stimulated macrophages, LPS-stimulated non-treated macrophages and LPS-stimulated vehicle-treated macrophages in the concentrations of IL-1β and TNF ($F=33.0$, $p<0.0001$ and $F=71.99$, $p<0.0001$, respectively, *Suppl. Fig. 1A,B*). No significant group differences were found between the latter groups in the measured parameters (IL-1β: $p=0.651$, TNF: $p=0.179$, Tukey's test) suggesting that alcohol solution alone does not affect LPS-induced release of the investigated pro-inflammatory cytokines.

A **B**

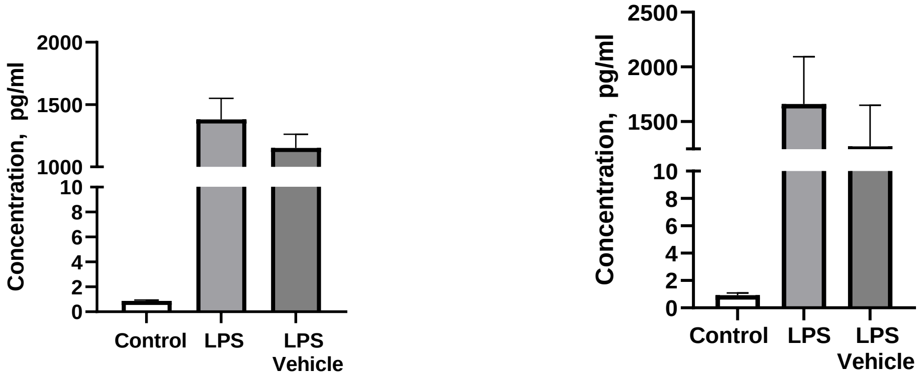

Supplementary Figure 1. Lack of effects of alcohol-containing vehicle on the cytokine release of LPS-stimulated human macrophages. (A) Concentrations of IL-1β were significantly elevated by LPS application where no group differences were found regardless the use of alcohol-containing vehicle **(B)** Similarly, concentrations of TNF were significantly increased by LPS application, no group differences were found regardless the use of alcohol-containing vehicle. Data presented as mean ±SEM.

Administration of resiquimod

Stock R848 (resiquimod, see the main text) was prepared by diluting in DMSO to a concentration of 10mg/mL. Stock resiquimod was then diluted 1:10 in sterile saline to 1mg/mL

and 200μL of the working solution was injected intraperitoneally (200μg of resiquimod per mouse). Control animals received sterile saline. All animals were culled 6 hours post-challenge.

‘Ceiling’ behavioral changes in mice challenged with 0.1 mg/kg of LPS

I order to determine an optimal dose of LPS injection in a mouse model of ‘sickness behavior’, a separate study was carried out using the novel cage test, marble test, forced swim model and the tail suspension paradigm. These experiments have shown ‘ceiling’ behavioral changes in mice injected with the dose of LPS of 0.1 mg/kg and studied 6 h thereafter (*Supplementary Fig.2*). There were significant group differences in the number of rearings in the novel cage (F=17.23, p<0.0001, one-way ANOVA), and, in comparison to non-challenged mice, significant decrease in this parameter in the LPS-non-treated mice (p<0.0001) and in IP-treated LPS-challenged animals p=0.0019, Tukey’s test, *Suppl. Fig.2A*). As for the number displaced pellets in the marble test (Veniaminova et al., 2021), significant group differences were found (F=4.93, p=0.029). In comparison to non-challenged mice, there was a significant decrease in this parameter in the LPS-non-treated mice (p=0.0291) and IP-treated LPS-challenged animals P=0.048, *Suppl. Fig.2B*). In the forced swim test, there was a significant group difference between the groups in the latency to float (F=10.34, p=0.0012). In comparison to non-challenged animals, the latency to float was significantly shorter in the LPS-non-treated mice (p=0.0018) and IP-treated LPS-challenged animals P=0.004, *Suppl. Fig.2C*). In this model, there was a significant group difference between the groups in the duration of floating (F=10.05, p=0.0013). In comparison to the non-challenged group, the duration of floating was significantly longer in the LPS-non-treated mice (p=0.0013) and IP-treated LPS-challenged animals P=0.0094, *Suppl. Fig.2D*). In the tail suspension paradigm, there were significant group differences in the duration of immobility (F=8.35, p=0.0039). In comparison to the non-challenged group, the duration of immobility was significantly longer in the LPS-non-treated mice (p=0.012) and IP-treated LPS-challenged animals p=0.0067, *Suppl. Fig.2E*).

A

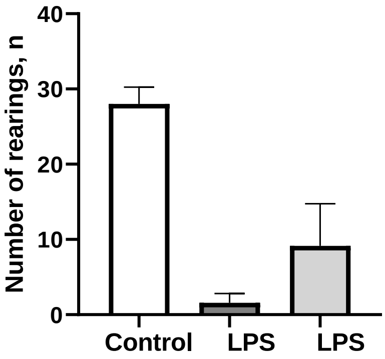

B

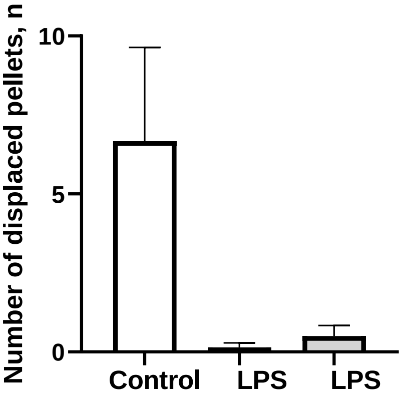

C D E

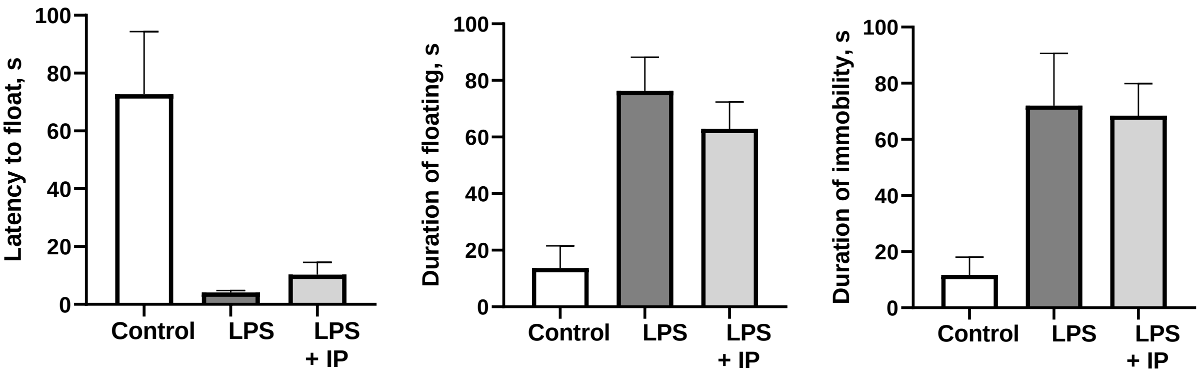

Supplementary Figure 2. The dose of LPS of 0.1 mg/kg has induced profound behavioural changes. (A) In the novel cage test, significant decrease in the number of rearings was found in the LPS-non-treated and IP-treated LPS-challenged mice in comparison with non-challenged controls. **(B)** In the marble test, the number of displaced pellets at 30th min of the test was significantly smaller in the LPS-non-treated and IP-treated LPS-challenged mice in comparison with non-challenged mice. In the forced swim test, in comparison to non-challenged animals there was a significant **(C)** decrease in the latency to float and **(D)** an increase in the duration of floating in both LPS-injected groups. **(E)** Similarly, the duration of immobility was significantly longer in the LPS-non-treated mice and IP-treated LPS-challenged animals than in the non-challenged group. Data presented as mean ±SEM.

Behavioural testing

Behavioural tests were carried out during the active period of the animals' light cycle (between 09:00–17:00); behaviour was recorded and scored offline. The experimenter was unaware of treatment each animal had received. Behavioural equipment was thoroughly cleaned with water between each test.

Novel Cage

The 5-min long novel cage test was carried out to assess exploration of a new environment as described elsewhere (Strekalova et al., 2004, 2015, Couch et al., 2016). Mice were introduced into a standard plastic cage (21 cm x 21 cm x 15 cm) filled with fresh sawdust. The number of exploratory rears was counted under red light per each minute, and summed up for minutes 1-5 of the test.

Open Field

In the open field model, mice were placed in a square white plastic box (45 x 45 x 45 cm) illuminated with white light (25 lx) near the wall for 10 min (Open Science, Moscow, Russia). The number of crossings of peripheral zone of the arena (0-10 cm from the wall) and remaining central area, a measure of anxiety-like behaviour were scored as described elsewhere (Pavlov et al., 2019; de Munter et al., 2021). The recordings were analysed using the automated video-tracking Viewpoint software (Viewpoint, Lyon, France).

Forced swim test

The test was carried out as described elsewhere (Malatynska et al., 2012). Animals were placed into a plastic transparent cylinder (Ø 17 cm) filled with water (+23°C, water height 13 cm, cylinder height 20 cm) under subtle lightning (light intensity 25 lx) for 6 min. Their parameters of floating behaviour that was defined by the absence of any directed movements of the animals' head and body. The total time spent floating was evaluated for the entire duration of the test, the latency to float was recorded as well using the automated video-tracking Viewpoint software (Viewpoint, Lyon, France).

Supplementary Table 2. Sequences of primers used for qRT-PCR

Primers were purchased from Sigma. Relative expression was determined by the 2-ΔΔCT method, normalized to GAPDH as the housekeeping gene (PrimerDesign).

Gene	Forward primer 5′–3′	Reverse primer 5′–3′
GAPDH	TGCACCACCAACTGCTTAG	GGATGCAGGGATGATGTTC
SAA-2	TGGCTGGAAAGATGGAGACAA	AAAGCTCTCTCTTGCATCACTG
ACE-2	GGGTGGTGCTGAAGTACGAT	CCAAAGACTGCTTTGCATCA
CXCL1	GCTGGGATTCACCTCAAGAAC	TGTGGCTATGACTTCGGTTTG
CXCL10	CATCCCGAGCCAACCTTCC	CACTCAGACCCAGCAGGAT
IL-1β	TGTAATGAAAGACGGCACACC	TCTTCTTTGGGTATTGCTTGG
IL-6	ACTCACCTCTTCAGAACGAATTG	CCATCTTTGGAAGGTTCAGGTTG

High-potency-liquid chromatography (HPLC)-high-resolution-mass-spectrometry (HRMS)

The analysis of herbal IP was carried out by the means of HPLC method followed by tandem mass spectrometry of high resolution (HPLC/MSHR). For the assay, an aliquot of 0.2 mL was placed to a centrifuge tube of 2.0 mL and 1.0 mL of ethanol was added. After shaking for 5 min, this mixture was centrifuged at the speed 10 000 / min for 5 min. Next, the solution was analyzed using the chromatographic system Agilent 1290 Infinity II (Agilent Technologies, Santa Clara, CA, USA), in conjunction with quadrupole-time streaming high precision mass detector Agilent 6545 Q-TOF LC/MS (Agilent Technologies, Santa Clara, CA, USA).

Chromatographic segregation was carried out using the column Zorbax Eclipse Plus C18 RRHD 2.1 mm × 50 mm × 1.8 µm (959757-902, Agilent Technologies, Santa Clara, CA, USA) and a 5 mm-pre-column (821725-901, Agilent Technologies, Santa Clara, CA, USA) under thermostat temperature of 35C°. For the fluent component phase, a 0.1% water solution of formic acid was used (solvent A) as well as an acetonitrile solution (solvent B). The speed of flow was 0.4 mL/min. The analysis was undertaken in a gradient regimen, a content of a solvent B was determined to be linear in a range from 5 to 100% till the 15th minute, the solution was kept under 100% B content till the 17th min, followed by a switch to a 5%-solvent B; a stabilization of

a fluent component was performed for another 2 min. The volume of the sample introduced was 1 μL.

The quadrupole-time streaming high precision mass detector was applied along with the source of ionization using electro spreading elements (Dual AJS ESI) under the conditions of ionization of positively and negatively charged ions. N2 was employed for a drying gas, under a temperature of 350C^o and the speed of a flow of 10 L/min. The temperature of additional overheating gas was 400 °C and the speed of a flow was 12 L/min, 90W fragmentation power. Under the conditions of mass spectrometry (MC), the quadrupole was tuned for streaming a full ion current, under conditions MC^2, the quadrupole has released only ion-precursor as a proteinized molecule of reference in a spectrum $\Delta m/z=1.3$ a.m. Dissociation spectra that were induced by strikes (DIS) that were measured under MC^2 conditions were obtained as a result of strikes of ion-precursor molecules against N2 molecules with energy calculated in a range: 10 - 50 eW. A range of scanning of mass was 100–1700 Da in MC regime and 30–700 Da in the MC^2 regime. The detector was functioning under conditions of enlarged dynamic range (EDR) of 2GHz, the speed of scanning was 1.5 spectra / sec. The tuning of the device and adjustment of the bore for a precision of mas definition was carried out automatically in real time using standard calibrating solutions, according to manufacturer instructions.

The identification of the components of investigated composition was carried out by the determination of exact mass and calculations of brutto-formula of the components, as well as by the use of previously obtained in a course of MC2 experiments DIS spectra and a comparison of these data against available standard data banks (NIST 17 MS /MS, Metlin AM PCD, MoNA MassBank GNPS).

Supplementary Table 3. Summary of mean and SEM for gene expression in liver

The analysis of relative fold expression of mRNA of immunomodulatory factors in the liver of mice with induced systemic inflammation has revealed the effects of inflammatory challenge and herbal IP (*Supplementary Table 3*; see also the main text and Fig.2).

**Groups**	**Genes**					
	**SAA-2**	**ACE-2**	**CXCL 1**	**CXCL 10**	**IL-1B**	**IL-6**
**Control**	1.0±0.32	1.0±0.23	1.0±0.23	1.0±0.12	1.0±0.08	1.0±0.19
**Resiquimod**	366.93±12.76	0.94±0.11	28.70±5.41	210.07±40,89	4.84±0.59	5.03±0.84
**IP**	1.96±0.74	0.92±0.06	2.25±1.39	1.21±0.42	1.19±0.12	1.04±0.14
**Resiquimod + IP**	348.04±22.59	0.64±0.07	23.16±3.29	114.25±16.80	4.44±0.30	4.53±0.71

A comparison of relative fold expression of mRNA of immunomodulatory factors in the spleen of mice with induced systemic inflammation has revealed the effects of inflammatory challenge and herbal IP (*Supplementary Table 4*; see also the main text and Fig.2).

Supplementary Table 4. Summary of mean and SEM for gene expression in spleen

Groups	Genes					
	SAA-2	ACE-2	CXCL 1	CXCL 10	IL-1B	IL-6
**Control**	1.0±0.6	1.0±0.3	1.0±0.04	1.0±0.04	1.0±0.26	1.0±0.014
**Resiquimod**	9.52±3.78	2.27±1.25	8.16±4.62	5.9±2.18	3.87±1.92	1.43±0.23
**IP**	82.60±5.77	2.19±0.32	0.94±0.03	0.88±0.02	0.97±0.1	1.0±0.02
**Resiquimod + IP**	143.39±8.63	235±0.24	2.01±0.43	5.53±1.04	2.44±0.48	1.10±0.04

Blood cell counts of mice with induced systemic inflammation were affected by inflammatory challenge and herbal IP (*Supplementary Table 5;* see also the main text and Fig.3).

Supplementary Table 5. Summary of mean and SEM for absolute data on the number of blood cells

Groups	Blood cells				
	Neutrophils	Monocytes	Eosinophils	Lymphocytes	Basophils
**Control**	1.09±0.15	0.18±0.05	0.6±0.18	1.3±0.18	0.08±0.02
**Resiquimod**	1.32±0.47	0.13±0.05	3.15±1.87	1.23±0.36	0.68±0.62
**IP**	1.78±0.25	0.19±0.08	1.31±1.44	1.14±0.2	0.05±0.01
**Resiquimod + IP**	0.47±0.10	0.05±0.01	2.28±0.55	0.87±0.15	0.06±0.01

HPLC-HRMS analysis of herbal IP and its physiological properties

Based on the methodology applied, the detection of negatively charged ions did not reveal any compounds. For mass-spectrochromogramm that was recorded for positively charged ions, the threshold of detection was set up as 50000 counts. 11 compounds were identified as constituents of herbal IP (*Supplementary Table 6*).

Supplementary Table 6. Chemical composition of IP: the main constituents

N	Retention time, min	$[M+H]^+$, measured	Name of compound	Brutto-formula	Relative content, %
1	0.40	104.1075	Choline	$C_5H_{13}NO$	11.5

2	0.87	132.1022	Isoleucine	$C_6H_{13}NO_2$	1.38
3	0.95	294.1549	*N*-Fructosyl or glucosyl isoleucine (leucine)	$C_{12}H_{23}NO_7$	4.47
4	1.42	166.0863	Phenylalanine	$C_9H_{11}NO_2$	1.75
5	2.16	291.1015	γ-Glutamyl-(S)-allyl-cysteine	$C_{11}H_{18}N_2O_5S$	4.68
6	2.27	205.0970	Tryptophan	$C_{11}H_{12}N_2O_2$	0.37
7	3.12	295.1293	Glu-Phe	$C_{14}H_{18}N_2O_5$	2.73
8	3.16	355.1022	Chlorogenic acid	$C_{16}H_{18}O_9$	2.17
9	3.42	395.1311	Syringin	$C_{15}H_{18}N_6O_7$	0.8
10	5.93	465.1022	Isoquercitin or Hyperoside	$C_{21}H_{20}O_{12}$	0.35
11	6.04	465.1020	Isoquercitin or Hyperoside	$C_{21}H_{20}O_{12}$	0.4

Reported physiological effects of the main constituents of IP

Utilizing World of Science (WoS) search engine, we conducted a comprehensive search of articles focusing on biological functions and, specifically, role in immunomodulation of herbal IP compounds. We used WoS search engine to find articles featuring search terms "biological role", "immunomodulation" or "inflammation" and a name of a chemical compound to identify these studies. We limited record types to journal articles and reviews, eliminating conference proceedings. The criteria set for the literature search covered the last 40 years of publication period. The results of this search summarizing the main physiological and immunomodulatory properties of IP constituents and key representative references are presented in a *Supplementary Table 7*.

Supplementary Table 7. Functions of IP chemical ingredients in the physiology and immunomodulation

Name of compound	Functions in physiology	Functions in immunomodulation
Choline	Key role in the synthesis of phospholipid components of cell membranes, and in the synthesis of plasma lipoproteins. It plays crucial role in acetylcholine synthesis (Hollenbeck, 2012), expression of the molecules regulating synaptic plasticity: G9a, Prmt1, Ahcy, Dnmt1, Mat2a (Blusztajn and Mellott, 2012; Craciunescu et al., 2003), exerts pro-proliferative effects in the dentate gyrus (Wong-Goodrich et al., 2008) potentially via the up-regulation of hippocampal expression of Insulin-like growth factor 2 (IGF2) (Cline et al.,	Anti-inflammatory and anti-oxidative stress effects in patients with asthma (Mehta et al., 2010). Higher choline levels were associated with protective effects on the fetal brain in humans (Chowdhurya, Pathakwhich, 2020) and promoted maturation, improved immune responses of the offspring in a rat (Lewis et al., 2016).

	2012, 2015).	
Isoleucine	Regulates glucose consumption and utilization via acting on the intestinal and muscular glucose transporters (Zhang et al., 2017), increases insulin receptor sensitivity and the browning of white adipose tissue (Ma et al., 2020); regenerative effects in the skin injury model, a counteraction of nitrosative stress (Nie et al., 2018), a suppression of H_2O_2-mediated oxidative stress (Katayama et al., 2007).	Induction of the expression of peptides regulating host innate and adaptive immunity (Gu et al., 2019), ameliorative effects on the parameters of oxidative stress: a decrease of malondialdehyde concentration, protein carbonyl content, and an increase of the activities of anti-oxidant defense enzymes: superoxide dismutase, catalase, and glutathione reductase (Zhao et al., 2014), a stimulation of the immune response via β-defensin (Mao et al., 2013).
N-Fructosyl or glucosyl isoleucine (leucine)	The pivotal regulator of protein folding, oligomerization and transport (Helenius and Aebi, 2000), the regulator of protein metabolism that counteracts protein degradation (Nair et al., 1992) and stimulates muscle protein synthesis, enhancement of insulin-receptor signaling in a muscle (Garlick et al., 2005), a promotion of somatic growth (Zhou et al., 2020), anti-stress effects (Koo and Howe, 2012).	An anti-inflammatory action under hyper-catabolic conditions that is mediated via the L-glutamine pathway (Cruzat et al., 2014), antioxidant and immune-regulating effects (Zhou et al., 2020).
Phenylalanine	Crucial role in the synthesis of proteins, catecholamines and melanin (DePietro, Fernstrom, 1999) due to its function of a precursor of the amino acid L-tyrosine Phenylethylamine that regulates lipid metabolism, increases extracellular levels of dopamine, modulates noradrenergic transmission and antagonizes GABA(B) receptor-mediated signaling, suppressing their inhibitory effects (Perkowski, Warpeha, 2019).	A regulation of immune response to viral infection, as shown in IFN-α – treated HCV patients (Murr et al., 2014) and implication in altered neuro-immune abnormalities and inflammatory responses in the patients with various neuropsychiatric disorders including major depressive disorder (Strasser et al., 2017); anti-inflammatory effects that are mediated via a suppression of the accumulation of lipids in the liver (Byun et al., 2017), a suppression of ROS (Chen et al., 2016; Oliva et al., 2020).
γ-Glutamyl-(S)-allyl-cysteine	Anti-oxidative stress effects during aging and under various pathological conditions that are associated with compromised free radical cellular scavenging (Colín-González et al., 2012), metal-chelating properties, inhibition the synthesis of glycation end products (AGEs) (Tan et al., 2015).	Stimulation of scavenging of free radicals and pro-oxidant species, induction of the antioxidant enzymes and Nrf2 factor, inhibition of pro-oxidant enzymes (Colín-González et al., 2012); an inhibition of the main protease of SARS-CoV-2 (Parashar et al., 2021).
Tryptophan	A precursor of serotonin, and thus, key roles in numerous physiological processes such as the sleep-wake cycle, appetite, mood, gut functions, and pain (Richard et al., 2009; Carneiro et al., 2018; Yamamoto et al., 2021).	Anti-inflammatory and anti-oxidative actions that is mediated via calcium-sensing receptor activation (Kim et al., 2010; Xu et al., 2017). A regulation of T-cell proliferation and anti-inflammatory effects via serotonin, a suppression of immune cells via downstream metabolites and pro-apoptotic mechanisms (Widner et al., 2000; Moffett, Namboodiri, 2003).
Glu-Phe	A suppression of the lipogenesis in	A suppression of the immune response to an

	hepatocytes and a regulation of hepatic lipid homeostasis (Lee et al., 2017).	immunization in mice (Babu, Maurer, 1984).
Chlorogenic acid	Pivotal roles in glucose and lipid metabolism and beneficial anti-diabetic, anti-carcinogenic, anti-inflammatory, antimicrobial, anti-obesity and mnemotropic effects (Tajik et al., 2017; Kumar et al., 2020), neuroprotective and anti-ischemic effects (Sato et al., 2011; Yun et al., 2012; Feng et al., 2016; Lee et al., 2020).	Powerful effects on the innate immunity through MDA5, TLR7 and NF-κB signaling pathways (Abaidullah et al., 2021), also via vascular modulation chemotaxis, macrophage activation, cytokine secretion, and immunoglobulin production (Bagdas et al., 2020), a suppression of a generation of pro-oxidant and pro-inflammatory factors SOD-2, IL-2, TNF, activation of antioxidant enzymes and anti-inflammatory cytokines: IL-4, IL-13 (Lee et al., 2020)
Syringin	The inhibition of adipocyte differentiation, adipogenesis, stimulation of lipid metabolism (Hossin et al., 2021) and of muscle glucose uptake and utilization in muscles, anti-diabetic effects (Niu et al., 2008).	Anti-inflammatory and neuroprotective properties via anti-FOXO3a /NF-κB activities (Tan et al., 2021), anti-allergic effect that is mediated by an in inhibition of TNF and IL-1β production and cytotoxic T cell proliferation (Cho et al., 2021; Song et al., 2010).
Isoquercitin	A stimulation of scavenging of reactive oxygen species (Hammer et al., 2007; Li et al., 2020) resulting in a reduced depletion of glutathione and an attenuation of the nitric oxide-induced lipid peroxidation (Jung et al., 2010); an anti-carcinogenic, anti-inflammatory, antiviral, antioxidant, and psychostimulant activities, anti-coagulation action, regulation of capillary permeability and stimulation of mitochondrial biogenesis (Li et al., 2016).	Antioxidant and anti-inflammatory effects that are mediated through direct scavenging of reactive oxygen/nitrogen species (ROS/RNS) and the Inhibition of prooxidant enzymes, prostaglandins and, cytokines production, an induction of antioxidant enzymes (Hammer et al., 2007; Valentová et al., 2014; Procházková et al., 2011; Kim et al., 2014). Beneficial immunomodulatory effects in patients suffering from rheumatoid arthritis, inflammatory bowel disease, multiple sclerosis, and systemic lupus erythematosus in humans or animal models that are mediated via the induction of Th-1 derived cytokine, IFN-γ and inhibition of Th-2 derived cytokine, IL-4 (Nair et al., 2002; Shen et al., 2021).
Hyperoside	Antioxidant, anti-hyperglycemic, anti-cancer, anti-inflammatory, and anticoagulant activities (Yang et al., 2021); anti-atherosclerosis and anti-stress mnemotropic effects (Gong et al., 2017).	Anti-inflammatory effects that are mediated by NLRP1-pathway (Ku et al., 2014), the NF-κB signaling mechanisms (Jin et al., 2016) and other LPS-induced inflammatory responses.

Reference list

Abaidullah, M., Peng, S., Song, X., Zou, Y., Li, L., Jia, R., Yin, Z., 2021. Chlorogenic acid is a positive regulator of MDA5, TLR7 and NF-κB signaling pathways mediated antiviral

responses against Gammacoronavirus infection. Int Immunopharmacol 96, 107671. doi: 10.1016/j.intimp.2021.107671.

Babu, U.M., Maurer, P.H., 1984. An unusual suppression of immune response: pretreatment with Poly(Glu60,Phe40) suppresses response to poly(Glu60,Phe40). Cell Immunol 86, 83-9. doi: 10.1016/0008-8749(84)90361-7.

Bagdas, D., Gul, Z., Meade, J.A., Cam, B., Cinkilic, N., Gurun, M.S., 2020. Pharmacologic Overview of Chlorogenic Acid and its Metabolites in Chronic Pain and Inflammation. Curr Neuropharmacol 18, 216-228. doi: 10.2174/1570159X17666191021111809.

Blusztajn, J.K., Mellott, T.J., 2012. Choline nutrition programs brain development via DNA and histone methylation. Cent Nerv Syst Agents Med Chem 12, 82-94. doi: 10.2174/187152412800792706.

Byun, K., Yoo, Y.C., Son, M., Lee, J., Jeong, G.B., Park, Y.M., Salekdeh, G.H., Lee, B., 2017. Advanced glycation end-products produced systemically and by macrophages: A common contributor to inflammation and degenerative diseases. Pharmacology and Therapeutics 177, 44–55. https://doi.org/10.1016/j.pharmthera.2017.02.030.

Carneiro, I.B.C., Toscano, A.E., Lacerda, D.C., da Cunha, M.S.B., de Castro, R.M., Deiró, T.C.B.J., Medeiros, J.M.B., 2018. L-tryptophan administration and increase in cerebral serotonin levels: Systematic review. Eur J Pharmacol 836, 129-135. doi: 10.1016/j.ejphar.2018.08.009.

Cenk, E., Schmutz, C., Pahlke, G., Oertel, A., Kollarova, J., Mock, H.P., Matros, A., Marko, D., 2021. Immunomodulatory Properties of Blackberry Anthocyanins in THP-1 Derived Macrophages. Int J Mol Sci 22, 10483. doi: 10.3390/ijms221910483.

Chen, Q., Man, C., Li, D., Tan, H., Xie, Y., Huang, J., 2016. Arogenate Dehydratase Isoforms Differentially Regulate Anthocyanin Biosynthesis in Arabidopsis thaliana. Molecular Plant 12, 1609–1619. https://doi.org/10.1016/j.molp.2016.09.010.

Cho, J.Y., Nam, K.H., Kim, A.R., Park, J., Yoo, E.S., Baik, K.U., Yu, Y.H., Park, M.H., 2001. In-vitro and in-vivo immunomodulatory effects of syringin. J Pharm Pharmacol 53, 1287-94. doi: 10.1211/0022357011776577.

Chowdhury, P., Pathak, P., 2020. Neuroprotective immunity by essential nutrient "Choline" for the prevention of SARS CoV2 infections: An in silico study by molecular dynamics approach. Chem Phys Lett 761, 138057. doi: 10.1016/j.cplett.2020.138057.

Cline, B.H., Costa-Nunes, J.P., Cespuglio, R., Markova, N., Santos, A.I., Bukhman, Y.V., Kubatiev, A., Steinbusch, H.W., Lesch, K.P., Strekalova, T., 2015. Dicholine succinate, the neuronal insulin sensitizer, normalizes behavior, REM sleep, hippocampal pGSK3 beta and mRNAs of NMDA receptor subunits in mouse models of depression. Front Behav Neurosci 26, 37. doi: 10.3389/fnbeh.2015.00037.

Cline, B.H., Steinbusch, H.W., Malin, D., Revishchin, A.V., Pavlova, G.V., Cespuglio, R., Strekalova, T., 2012. The neuronal insulin sensitizer dicholine succinate reduces stress-induced depressive traits and memory deficit: possible role of insulin-like growth factor 2. BMC Neurosci 18, 110. doi: 10.1186/1471-2202-13-110.

Colín-González, A.L., Santana, R.A., Silva-Islas, C.A., Chánez-Cárdenas, M.E., Santamaría, A., Maldonado, P.D., 2012. The antioxidant mechanisms underlying the aged garlic extract- and S-allylcysteine-induced protection. Oxid Med Cell Longev 2012, 907162. doi: 10.1155/2012/907162.

Couch, Y., Trofimov, A., Markova, N., Nikolenko, V., Steinbusch, H.W., Chekhonin, V., Schroeter, C., Lesch, K.P., Anthony, D.C., Strekalova, T., 2016. Low-dose lipopolysaccharide (LPS) inhibits aggressive and augments depressive behaviours in a chronic mild stress model in mice. J Neuroinflammation 13, 108. doi: 10.1186/s12974-016-0572-0.

Craciunescu, C.N., Albright, C.D., Mar, M.H., Song, J., Zeisel, S.H., 2003. Choline availability during embryonic development alters progenitor cell mitosis in developing mouse hippocampus. J Nutr 133, 3614-8. doi: 10.1093/jn/133.11.3614.

Cruzat, V.F., Krause, M., Newsholme, P., 2014. Amino acid supplementation and impact on immune function in the context of exercise. J Int Soc Sports Nutr 14, 61. doi: 10.1186/s12970-014-0061-8.
De Munter, J., Lyundup, A., Pavlov, D., Walters, E., Gorlova, A., Veniaminova, E., Umrukhin, A., Kalueff, A., Svistunov, A., Kramer, B., Lesch, K.P., Strekalova, T., 2020. Neuro-Cell therapy improves motor outcomes and suppresses inflammation during experimental syndrome of amyotrophic lateral sclerosis in mice. CNS Neuroscience & Therapeutics 00, 1-14. doi: 10.1111/cns.13280.
De Munter, J., Pavlov, D., Gorlova, A., Sicker, M., Proshin, A., Kalueff, A.V., Svistunov, A., Kiselev, D., Nedorubov, A., Morozov, S., Umriukhin, A., Lesch, K.P., Strekalova, T., Schroeter, C.A., 2021. Increased Oxidative Stress in the Prefrontal Cortex as a Shared Feature of Depressive- and PTSD-Like Syndromes: Effects of a Standardized Herbal Antioxidant. Front Nutr 8, 661455. doi: 10.3389/fnut.2021.661455.
DePietro, F.R., Fernstrom, J.D., 1999. The relative roles of phenylalanine and tyrosine as substrates for DOPA synthesis in PC12 cells. Brain Res 831, 72-84. doi: 10.1016/s0006-8993(99)01400-6.
Batiha, G. E. S., Beshbishy, A. M., Wasef, L. G., Elewa, Y. H. A., Al-Sagan, A. A., El-Hack, M. E. A., Taha, A. E., Abd-Elhakim, Y. M., Devkota, H. P., 2020. Chemical constituents and pharmacological activities of garlic (Allium sativum L.): A review. Nutrients 12. https://doi.org/10.3390/nu12030872
Feng, Y., Yu, Y. H., Wang, S. T., Ren, J., Camer, D., Hua, Y. Z., Zhang, Q., Huang, J., Xue, D. L., Zhang, X. F., Huang, X. F., Liu, Y., 2016. Chlorogenic acid protects d-galactose-induced liver and kidney injury via antioxidation and anti-inflammation effects in mice. Pharmaceutical Biology 54, 1027–1034.https://doi.org/10.3109/13880209.2015.1093510.
Garlick, P.J., 2005. The role of leucine in the regulation of protein metabolism. J Nutr 135, 1553S-6S. https://doi.org/10.1093/jn/135.6.1553S.
Gong, Y., Yang, Y., Chen, X., Yang, M., Huang, D., Yang, R., Zhou, L., Li, C., Xiong, Q., Xiong, Z., 2017. Hyperoside protects against chronic mild stress-induced learning and memory deficits. Biomed Pharmacother 91, 831-840. doi: 10.1016/j.biopha.2017.05.019.
Gu, C., Mao, X., Chen, D., Yu, B., Yang, Q., 2019. Isoleucine Plays an Important Role for Maintaining Immune Function. Curr Protein Pept Sci 20, 644-651. doi: 10.2174/1389203720666190305163135.
Hammer, K.D., Hillwig, M.L., Solco, A.K., Dixon, P.M., Delate, K., Murphy, P.A., Wurtele, E.S., Birt, D.F., 2007. Inhibition of prostaglandin E(2) production by anti-inflammatory hypericum perforatum extracts and constituents in RAW264.7 Mouse Macrophage Cells. J Agric Food Chem 55, 7323-31. doi: 10.1021/jf0710074.
Han, X., Parker, T.L., 2017. Antiinflammatory Activity of Cinnamon (Cinnamomum zeylanicum) Bark Essential Oil in a Human Skin Disease Model. Phytother Res 31, 1034-1038. doi: 10.1002/ptr.5822.
Helenius, A., Aebi, M., 2000. lntracellular Functions of N-Linked Clycans. In J. R. Wieser. A. Heisner, P. Stehling, F. Oesch. W. Reutter. FEES Lett (Vol. 1).
Hollenbeck, C.B., 2012. An introduction to the nutrition and metabolism of choline. Cent Nerv Syst Agents Med Chem 12, 100-13. doi: 10.2174/187152412800792689.
Hossin, A.Y., Inafuku, M., Takara, K., Nugara, R.N., Oku, H., 2021. Syringin: A Phenylpropanoid Glycoside Compound in Cirsium brevicaule A. GRAY Root Modulates Adipogenesis. Molecules 26, 1531. doi: 10.3390/molecules26061531.
Jeong, Y. Y., Ryu, J. H., Shin, J. H., Kang, M. J., Kang, J. R., Han, J., & Kang, D., 2016. Comparison of anti-oxidant and anti-inflammatory effects between fresh and aged black garlic extracts. Molecules 21. https://doi.org/10.3390/molecules21040430
Jin, X.N., Yan, E.Z., Wang, H.M., Sui, H.J., Liu, Z., Gao, W., Jin Y., 2016. Hyperoside exerts anti-inflammatory and anti-arthritic effects in LPS-stimulated human fibroblast-like

synoviocytes in vitro and in mice with collagen-induced arthritis. Acta Pharmacol Sin 37, 674-86. doi: 10.1038/aps.2016.7.
Jung, S.H., Kim, B.J., Lee, E.H., Osborne, N.N., 2010. Isoquercitrin is the most effective antioxidant in the plant Thuja orientalis and able to counteract oxidative-induced damage to a transformed cell line (RGC-5 cells). Neurochem Int 7, 713-21. doi: 10.1016/j.neuint.2010.08.005.
Katayama, S., Ishikawa, S. I., Fan, M. Z., Mine, Y., 2007. Oligophosphopeptides derived from egg yolk phosvitin up-regulate γ-glutamylcysteine synthetase and antioxidant enzymes against oxidative stress in Caco-2 cells. Journal of Agricultural and Food Chemistry 55, 2829–2835. https://doi.org/10.1021/jf0628936.
Kim, C.J., Kovacs-Nolan, J.A., Yang, C., Archbold, T., Fan, M.Z., Mine, Y., 2010. l-Tryptophan exhibits therapeutic function in a porcine model of dextran sodium sulfate (DSS)-induced colitis. J Nutr Biochem 21, 468-75. doi: 10.1016/j.jnutbio.2009.01.019.
Kim, D.H., Sung, B., Kang, Y.J., Jang, J.Y., Hwang, S.Y., Lee, Y., Kim, M., Im, E., Yoon, J.H., Kim, C.M., Chung, H.Y., & Kim, N.D., 2014. Anti-inflammatory effects of betaine on AOM/DSS-induced colon tumorigenesis in ICR male mice. International Journal of Oncology 45, 1250–1256. https://doi.org/10.3892/ijo.2014.2515.
Koo, A.J., Howe, G.A., 2012. Catabolism and deactivation of the lipid-derived hormone jasmonoyl-isoleucine. Front Plant Sci 3, 19. doi: 10.3389/fpls.2012.00019.
Ku, S.K., Kwak, S., Kwon, O.J., Bae, J.S., 2014. Hyperoside inhibits high-glucose-induced vascular inflammation in vitro and in vivo. Inflammation 37: 1389-400. doi: 10.1007/s10753-014-9863-8.
Kumar, R., Sharma, A., Iqbal, M.S., Srivastava, J.K., 2020. Therapeutic Promises of Chlorogenic Acid with Special Emphasis on its Anti-Obesity Property. Curr Mol Pharmacol 13, 7-16. doi: 10.2174/1874467212666190716145210.
Lee, T.K., Kang, I.J., Kim, B., Sim, H.J., Kim, D.W., Ahn, J.H., Lee, J.C., Ryoo, S., Shin, M.C., Cho, J.H., Kim, Y.M., Park, J.H., Choi, S.Y., Won, M.H., 2020. Experimental Pretreatment with Chlorogenic Acid Prevents Transient Ischemia-Induced Cognitive Decline and Neuronal Damage in the Hippocampus through Anti-Oxidative and Anti-Inflammatory Effects. Molecules 25, 3578. doi: 10.3390/molecules25163578.
Lee, Y.G., Cho, J.Y., Hwang, E.J., Jeon, T.I., Moon, J.H., 2017. Glu-Phe from onion (Allium Cepa L.) attenuates lipogenesis in hepatocytes. Biosci Biotechnol Biochem 81, 1409-1416. doi: 10.1080/09168451.2017.1303358.
Lewis, E.D., Richard, C., Goruk, S., Dellschaft, N.S., Curtis, J.M., Jacobs, R.L., Field C.J., 2016. The Form of Choline in the Maternal Diet Affects Immune Development in Suckled Rat Offspring. J Nutr 146, 823-30. doi: 10.3945/jn.115.225888.
Li, X., Jiang, Q., Wang, T., Liu, J., Chen, D., 2016. Comparison of the Antioxidant Effects of Quercitrin and Isoquercitrin: Understanding the Role of the 6″-OH Group. Molecules 21, 1246. doi: 10.3390/molecules21091246.
Li, Y., Yao, J., Han, C., Yang, J., Chaudhry, M.T., Wang, S., Liu, H., Yin, Y., 2016. Quercetin, Inflammation and Immunity. Nutrients 15, 167. doi: 10.3390/nu8030167.
Ma, Q., Zhou, X., Hu, L., Chen, J., Zhu, J., Shan, A., 2020. Leucine and isoleucine have similar effects on reducing lipid accumulation, improving insulin sensitivity and increasing the browning of WAT in high-fat diet-induced obese mice. Food Funct 26, 2279-2290. doi: 10.1039/c9fo03084k.
Malatynska, E., Steinbusch, H.W.M., Redkozubova, O., Bolkunov, A., Kubatiev, A., Yeritsyan, N.B., Vignisse, J., Bachurin, S., Strekalova, T., 2012. Anhedonic-like traits and lack of affective deficits in 18-month-old C57BL/6 mice: Implications for modeling elderly depression. Exp Gerontol 47, 552–564. https://doi.org/10.1016/j.exger.2012.04.010
Mao, X., Qi, S., Yu, B., He, J., Yu, J., Chen, D., 2013. Zn(2+) and L-isoleucine induce the expressions of porcine β-defensins in IPEC-J2 cells. Mol Biol Rep 40, 1547-52. doi: 10.1007/s11033-012-2200-0.

Mehta, A.K., Singh, B.P., Arora, N., Gaur, S.N., 2010. c Immunobiology 215: 527-34. doi: 10.1016/j.imbio.2009.09.004.
Moffett, J.R., Namboodiri, M.A., 2003. Tryptophan and the immune response. Immunol Cell Biol 81: 247-65. doi: 10.1046/j.1440-1711.2003.t01-1-01177.x.
Murr, C., Grammar, T.B., Meinitzer, A., Kleber, M.E., März, W., Fuchs, D., 2014. Immune activation and inflammation in patients with cardiovascular disease are associated with higher phenylalanine to tyrosine ratios: the ludwigshafen risk and cardiovascular health study. J Amino Acids 783730. doi: 10.1155/2014/783730.
Nair, K.S., Schwartz, R.G., Welle, S., 1992. Leucine as a regulator of whole body and skeletal muscle protein metabolism in humans. Am J Physiol 263, E928-34. doi: 10.1152/ajpendo.1992.263.5.E928.
Nair, M.P.N., Kandaswami, C., Mahajan, S., Chadha, K.C., Chawda, R., Nair, H., Kumar, N., Nair, R.E., Schwartz, S.A., 2002. The flavonoid, quercetin, differentially regulates Th-1 (IFNg) and Th-2 (IL4) cytokine gene expression by normal peripheral blood mononuclear cells. Biochim. Biophys. Acta 1593, 29–36. doi: 10.1016/s0167-4889(02)00328-2.
Nascimento, S.S.D.C., de Queiroz, J.L.C., de Medeiros, A.F., de França Nunes, A.C., Piuvezam, G., Maciel, B.L.L., Passos, T.S., de Araújo Morais, A.H., 2021. What are the mechanisms of action of anti-inflammatory agents in adipose tissue?: A protocol for systematic review and meta-analysis. Medicine (Baltimore) 100, e24677. doi: 10.1097/MD.0000000000024677.
Nie, C., He, T., Zhang, W., Zhang, G., Ma, X., 2018. Branched Chain Amino Acids: Beyond Nutrition Metabolism. Int J Mol Sci 19, 954. doi: 10.3390/ijms19040954.
Niu, H.S., Liu, I.M., Cheng, J.T., Lin, C.L., Hsu, F.L., 2008. Hypoglycemic effect of syringin from Eleutherococcus senticosus in streptozotocin-induced diabetic rats. Planta Med 74, 109-13. doi: 10.1055/s-2008-1034275.
Nunes, C.D.R., Barreto, Arantes M., Menezes de Faria Pereira, S., Leandro da Cruz, L., de Souza Passos, M., Pereira de Moraes, L., Vieira, I.J.C., Barros de Oliveira, D., 2020. Plants as Sources of Anti-Inflammatory Agents. Molecules 25, 3726. doi: 10.3390/molecules25163726.
Oliva, M., Hatan, E., Kumar, V., Galsurker, O., Nisim-Levi, A., Ovadia, R., Galili, G., Lewinsohn, E., Elad, Y., Alkan, N., Oren-Shamir, M., 2020. Increased phenylalanine levels in plant leaves reduces susceptibility to Botrytis cinerea. Plant Science 290. https://doi.org/10.1016/j.plantsci.2019.110289.
Ortiz, M.I., Cariño-Cortés, R., Ponce-Monter, H.A., González-García, M.P., Castañeda-Hernández, G., Salinas-Caballero, M., 2017. Synergistic Interaction of Matricaria Chamomilla Extract with Diclofenac and Indomethacin on Carrageenan-Induced Paw Inflammation in Rats. Drug Development Research, 78, 360–367. https://doi.org/10.1002/ddr.21401.
Paczkowska-Walendowska, M., Gościniak, A., Szymanowska, D., Szwajgier, D., Baranowska-Wójcik, E., Szulc, P., Dreczka, D., Simon, M., Cielecka-Piontek, J., 2021. Blackberry Leaves as New Functional Food? Screening Antioxidant, Anti-Inflammatory and Microbiological Activities in Correlation with Phytochemical Analysis. Antioxidants (Basel) 10, 1945. doi: 10.3390/antiox10121945.
Parashar, A., Shukla, A., Sharma, A., Behl, T., Goswami, D., Mehta, V., 2021. Reckoning γ-Glutamyl-S-allylcysteine as a potential main protease (mpro) inhibitor of novel SARS-CoV-2 virus identified using docking and molecular dynamics simulation. Drug Dev Ind Pharm 47, 699-710. doi: 10.1080/03639045.2021.1934857.
Pavlov, D., Bettendorff, L., Gorlova, A., Olkhovik, A., Kalueff, A.V., Ponomarev, E.D., Inozemtsev, A., Chekhonin, V., Lesch, K.-P., Anthony, D.C., Strekalova, T., 2019. Neuroinflammation and aberrant hippocampal plasticity in a mouse model of emotional stress evoked by exposure to ultrasound of alternating frequencies. Prog

Neuropsychopharmacol Biol Psychiatry 90, 104–116. https://doi.org/10.1016/j.pnpbp.2018.11.014

Perkowski, M.C., Warpeha, K.M., 2019. Phenylalanine roles in the seed-to-seedling stage: Not just an amino acid. Plant Sci 289, 110223. doi: 10.1016/j.plantsci.2019.110223.

Procházková, D., Boušová, I., Wilhelmová, N., 2011. Antioxidant and prooxidant properties of flavonoids. Fitoterapia 82, 513–523. https://doi.org/10.1016/j.fitote.2011.01.018.

Richard, D.M., Dawes, M.A., Mathias, C.W., Acheson, A., Hill-Kapturczak, N., Dougherty, D.M., 2009. L-Tryptophan: Basic Metabolic Functions, Behavioral Research and Therapeutic Indications. Int J Tryptophan Res 23, 45-60. doi: 10.4137/ijtr.s2129.

Sato, Y., Itagaki, S., Kurokawa, T., Ogura, J., Kobayashi, M., Hirano, T., Sugawara, M., Iseki, K., 2011. In vitro and in vivo antioxidant properties of chlorogenic acid and caffeic acid. Int J Pharm 403, 136-8. doi: 10.1016/j.ijpharm.2010.09.035.

Shen, P., Lin, W., Deng, X., Ba, X., Han, L., Chen, Z., Qin, K., Huang, Y., Tu, S., 2021. Potential Implications of Quercetin in Autoimmune Diseases. Front Immunol 12, 689044. doi: 10.3389/fimmu.2021.689044.

Song, Y.Y., Li, Y., Zhang, H.Q., 2010. Therapeutic effect of syringin on adjuvant arthritis in rats and its mechanisms. 45, 1006-11.

Strasser, B., Sperner-Unterweger, B., Fuchs, D., Gostner, J.M., 2017. Mechanisms of Inflammation-Associated Depression: Immune Influences on Tryptophan and Phenylalanine Metabolisms. Curr Top Behav Neurosci 31, 95-115. doi: 10.1007/7854_2016_23. PMID: 27278641.

Strekalova, T., Evans, M., Chernopiatko, A., Couch, Y., Costa-Nunes, J., Cespuglio, R., Chesson, L., Vignisse, J., Steinbusch, H.W., Anthony, D.C., Pomytkin, I., Lesch, K.-P., 2015. Deuterium content of water increases depression susceptibility: the potential role of a serotonin-related mechanism. Behav Brain Res 277, 237–244. https://doi.org/10.1016/j.bbr.2014.07.039.

Strekalova, T., Spanagel, R., Bartsch, D., Henn, F.A., Gass, P., 2004. Stress-induced anhedonia in mice is associated with deficits in forced swimming and exploration. Neuropsychopharmacology. 11, 2007-17. doi: 10.1038/sj.npp.1300532.

Tai, A., Sawano, T., Yazama, F., Ito, H., 2011. Evaluation of antioxidant activity of vanillin by using multiple antioxidant assays. Biochim Biophys Acta 1810, 170-7. doi: 10.1016/j.bbagen.2010.11.004.

Tajik, N., Tajik, M., Mack, I., Enck, P., 2017. The potential effects of chlorogenic acid, the main phenolic components in coffee, on health: a comprehensive review of the literature. Eur J Nutr. 56, 2215-2244. doi: 10.1007/s00394-017-1379-1.

Tan, D., Zhang, Y., Chen, L., Liu, L., Zhang, X., Wu, Z., Bai, B., Ji, S., 2015. Decreased glycation and structural protection properties of γ-glutamyl-S-allyl-cysteine peptide isolated from fresh garlic scales (Allium sativum L.). Nat Prod Res 29, 2219-22. doi: 10.1080/14786419.2014.1003065.

Tan, J., Luo, J., Meng, C., Jiang, N., Cao, J., Zhao, J., 2021. Syringin exerts neuroprotective effects in a rat model of cerebral ischemia through the FOXO3a/NF-κB pathway. Int 90, 107268. doi: 10.1016/j.intimp.2020.107268.

Valentová, K., Vrba, J., Bancířová, M., Ulrichová, J., Křen, V., 2014. Isoquercitrin: pharmacology, toxicology, and metabolism. Food Chem Toxicol 68, 267-82. doi: 10.1016/j.fct.2014.03.018.

Vogl, S., Picker, P., Mihaly-Bison, J., Fakhrudin, N., Atanasov, A.G., Heiss, E.H., Wawrosch, C., Reznicek, G., Dirsch, V.M., Saukel, J., Kopp, B., 2013. Ethnopharmacological in vitro studies on Austria's folk medicine--an unexplored lore in vitro anti-inflammatory activities of 71 Austrian traditional herbal drugs. J Ethnopharmacol 149, 750-71. doi: 10.1016/j.jep.2013.06.007.

Widner, B., Leblhuber, F., Walli, J., Tilz, G.P., Demel, U., Fuchs, D., 2000. Tryptophan degradation and immune activation in Alzheimer's disease. J Neural Transm (Vienna) 107, 343-53. doi: 10.1007/s007020050029.

Wieland, L.S., Piechotta, V., Feinberg, T., Ludeman, E., Hutton, B., Kanji, S., Seely, D., Garritty, C., 2021. Elderberry for prevention and treatment of viral respiratory illnesses: a systematic review. BMC Complement Med Ther 21, 112. doi: 10.1186/s12906-021-03283-5.

Wong-Goodrich, S.J., Mellott, T.J., Glenn, M.J., Blusztajn, J.K., Williams, C.L., 2008. Prenatal choline supplementation attenuates neuropathological response to status epilepticus in the adult rat hippocampus. Neurobiol Dis 30, 255-69. doi: 10.1016/j.nbd.2008.01.008.

Xia, L., Shi, Y., Su, J., Friedemann, T., Tao, Z., Lu, Y., Ling, Y., Lv, Y., Zhao, R., Geng, Z., Cui, X., Lu, H., Schröder, S., 2021. Shufeng Jiedu, a promising herbal therapy for moderate COVID-19:Antiviral and anti-inflammatory properties, pathways of bioactive compounds, and a clinical real-world pragmatic study. Phytomedicine 85, 153390. doi: 10.1016/j.phymed.2020.153390.

Xu, C., Mathews, A.E., Rodrigues, C., Eudy, B.J., Rowe, C.A., O'Donoughue, A., Percival, S.S., 2018. Aged garlic extract supplementation modifies inflammation and immunity of adults with obesity: A randomized, double-blind, placebo-controlled clinical trial. Clin Nutr ESPEN 24, 148-155. doi: 10.1016/j.clnesp.2017.11.010.

Xu, N., Chen, G., Liu, H., 2017. Antioxidative categorization of twenty amino acids based on experimental evaluation. Molecules 22. https://doi.org/10.3390/molecules22122066.

Yamamoto, T., Azechi, H., Board, M., 2012. Essential role of excessive tryptophan and its neurometabolites in fatigue. Can J Neurol Sci. 39, 40-7. doi: 10.1017/s031716710001266x.

Yang, Y., Li, J., Rao, T., Fang, Z., Zhang, J., 2021. The role and mechanism of hyperoside against myocardial infarction in mice by regulating autophagy via NLRP1 inflammation pathway. J Ethnopharmacol 276, 114187. doi: 10.1016/j.jep.2021.114187.

Yun, N., Kang, J.W., Lee, S.M., 2012. Protective effects of chlorogenic acid against ischemia/reperfusion injury in rat liver: molecular evidence of its antioxidant and anti-inflammatory properties. J Nutr Biochem 23, 1249-55. doi: 10.1016/j.jnutbio.2011.06.018.

Zare, E., Alirezaei, A., Bakhtiyari, M., & Mansouri, A., 2019. Evaluating the effect of garlic extract on serum inflammatory markers of peritoneal dialysis patients: A randomized double-blind clinical trial study. BMC Nephrology 20. https://doi.org/10.1186/s12882-019-1204-6.

Zhang, S., Zeng, X., Ren, M., Mao, X., Qiao, S., 2017. Novel metabolic and physiological functions of branched chain amino acids: a review. J Anim Sci Biotechnol 23, 8:10. doi: 10.1186/s40104-016-0139-z.

Zhao, J., Feng, L., Liu, Y., Jiang, W., Wu, P., Jiang, J., Zhang, Y., Zhou X., 2014. Effect of dietary isoleucine on the immunity, antioxidant status, tight junctions and microflora in the intestine of juvenile Jian carp (Cyprinus carpio var. Jian). Fish Shellfish Immunol 41, 663-73. doi: 10.1016/j.fsi.2014.10.002.

Zhou, C., Lin, H., Huang, Z., Wang, J., Wang, Y., Yu, W., 2020. Effects of dietary leucine levels on intestinal antioxidant status and immune response for juvenile golden pompano (Trachinotus ovatus) involved in Nrf2 and NF-κB signaling pathway. Fish Shellfish Immunol 107, 336-345. doi: 10.1016/j.fsi.2020.10.012.
